# Embryonic origin of two ASD subtypes of social symptom severity: the larger the brain cortical organoid size, the more severe the social symptoms

**DOI:** 10.1186/s13229-024-00602-8

**Published:** 2024-05-25

**Authors:** Eric Courchesne, Vani Taluja, Sanaz Nazari, Caitlin M. Aamodt, Karen Pierce, Kuaikuai Duan, Sunny Stophaeros, Linda Lopez, Cynthia Carter Barnes, Jaden Troxel, Kathleen Campbell, Tianyun Wang, Kendra Hoekzema, Evan E. Eichler, Joao V. Nani, Wirla Pontes, Sandra Sanchez Sanchez, Michael V. Lombardo, Janaina S. de Souza, Mirian A. F. Hayashi, Alysson R. Muotri

**Affiliations:** 1 Autism Center of Excellence, Department of Neurosciences, University of California, San Diego, 8110 La Jolla Shores Dr., La Jolla, CA 92037 USA; 2 Department of Medical Genetics, Center for Medical Genetics, Peking University Health Science Center, Beijing, 100191 China; 3 Neuroscience Research Institute, Peking University, Key Laboratory for Neuroscience, Ministry of Education of China and National Health Commission of China, Beijing, 100191 China; 4 Department of Genome Sciences, University of Washington School of Medicine, Seattle, WA 98195 USA; 5 Howard Hughes Medical Institute, University of Washington, Seattle, WA 98195 USA; 6 Department of Pediatrics and Department of Molecular and Cellular Medicine, University of California, San Diego, Gilman Drive, La Jolla, CA 92093 USA; 7 Department of Pharmacology, Escola Paulista de Medicina (EPM), Universidade Federal de São Paulo (UNIFESP), São Paulo, SP Brazil; 8 Rady Children’s Hospital, Center for Academic Research and Training in Anthropogeny (CARTA), Archealization Center (ArchC), Kavli Institute for Brain and Mind, La Jolla, CA USA; 9 Laboratory for Autism and Neurodevelopmental Disorders, Center for Neuroscience and Cognitive Systems, Istituto Italiano di Tecnologia, Rovereto, Italy

## Abstract

**Background:**

Social affective and communication symptoms are central to autism spectrum disorder (ASD), yet their severity differs across toddlers: Some toddlers with ASD display improving abilities across early ages and develop good social and language skills, while others with “profound” autism have persistently low social, language and cognitive skills and require lifelong care. The biological origins of these opposite ASD social severity subtypes and developmental trajectories are not known.

**Methods:**

Because ASD involves early brain overgrowth and excess neurons, we measured size and growth in 4910 embryonic-stage brain cortical organoids (BCOs) from a total of 10 toddlers with ASD and 6 controls (averaging 196 individual BCOs measured/subject). In a 2021 batch, we measured BCOs from 10 ASD and 5 controls. In a 2022 batch, we  tested replicability of BCO size and growth effects by generating and measuring an independent batch of BCOs from 6 ASD and 4 control subjects. BCO size was analyzed within the context of our large, one-of-a-kind social symptom, social attention, social brain and social and language psychometric normative datasets ranging from N = 266 to N = 1902 toddlers. BCO growth rates were examined by measuring size changes between 1- and 2-months of organoid development. Neurogenesis markers at 2-months were examined at the cellular level. At the molecular level, we measured activity and expression of Ndel1; Ndel1 is a prime target for cell cycle-activated kinases; known to regulate cell cycle, proliferation, neurogenesis, and growth; and known to be involved in neuropsychiatric conditions.

**Results:**

At the BCO level, analyses showed BCO size was significantly enlarged by 39% and 41% in ASD in the 2021 and 2022 batches. The larger the embryonic BCO size, the more severe the ASD social symptoms. Correlations between BCO size and social symptoms were r = 0.719 in the 2021 batch and r = 0. 873 in the replication 2022 batch. ASD BCOs grew at an accelerated rate nearly 3 times faster than controls. At the cell level, the two largest ASD BCOs had accelerated neurogenesis. At the molecular level, Ndel1 activity was highly correlated with the growth rate and size of BCOs. Two BCO subtypes were found in ASD toddlers: Those in one subtype had very enlarged BCO size with accelerated rate of growth and neurogenesis; a profound autism clinical phenotype displaying severe social symptoms, reduced social attention, reduced cognitive, very low language and social IQ; and substantially altered growth in specific cortical social, language and sensory regions. Those in a second subtype had milder BCO enlargement and milder social, attention, cognitive, language and cortical differences.

**Limitations:**

Larger samples of ASD toddler-derived BCO and clinical phenotypes may reveal additional ASD embryonic subtypes.

**Conclusions:**

By embryogenesis, the biological bases of two subtypes of ASD social and brain development—profound autism and mild autism—are already present and measurable and involve dysregulated cell proliferation and accelerated neurogenesis and growth. The larger the embryonic BCO size in ASD, the more severe the toddler’s social symptoms and the more reduced the social attention, language ability, and IQ, and the more atypical the growth of social and language brain regions.

**Supplementary Information:**

The online version contains supplementary material available at 10.1186/s13229-024-00602-8.

## Background

Social affective and communication symptoms are central to autism spectrum disorder (ASD), yet their severity differs across toddlers with the disorder: Some ASD toddlers display improving abilities across early ages and develop good language skills and social relationships, while others with profound autism have persistently low social and cognitive skills, may be non-verbal, have few if any friends, and require lifelong care [2, 3]. The biological origins of these opposite ASD social severity subtypes and developmental trajectories are not known. As such, there is an urgent need to discover biological origins of social subtypes at the youngest ages possible as a pathway towards precision medicine.

While genetic, postmortem and cell model studies show ASD is a prenatal, progressive disorder involving multiple stages and processes [4, 5], it is a remarkable fact that none of these studies have demonstrated a within-child correlation between any prenatal stage or process and the severity of an ASD child’s social symptoms. Moreover, studies have not identified prenatal biological features indicative of social and neural subtypes in ASD toddlers at the age of first symptom presentation. While social and neural subtypes have been found in ASD toddlers by data-driven, unsupervised patient subtyping methods, the biological origins of subtypes are unknown and could be a complex interweaving of genetic and environmental factors across prenatal and postnatal developmental time periods.

Possible prenatal developmental origins of core social variation in autism are largely unstudied. The reasons for this lack of identification of prenatal correlates of ASD social severity and social heterogeneity are multiple and understandable. For example, there is no direct, in vivo means to know whether a fetus will develop ASD and no method of assaying potentially relevant prenatal features even if an ASD outcome were to be suspected. As another example, since ASD social symptoms vary widely, small sample postmortem studies inadequately represent that social heterogeneity and are impoverished in information about early-age social phenotype. As another example, developmental animal studies inadequately model typical and ASD social communication and social cognition. Fortunately, ASD patient-derived iPSC models provide a path towards gaining insight into prenatal pathobiology that can be correlated within subject with ASD social symptoms. To date, however, this has not been done. Here we take this path in an a priori designed study.

The power of patient-based iPSC comes from properly choosing patients whose disease symptoms are to be specifically elucidated by targeted iPSC models and measures. In that way, iPSC results can be meaningfully related to the specific disease feature at issue. Oddly, this has never been done for social symptoms in ASD, despite that fact that social communication and its variation are at the center of the disorder. Among more than 90 published ASD iPSC model studies (Supplemental Tables S1-S2), study procedures did not ascertain, recruit, clinically and diagnostically phenotype, longitudinally follow and neural and behaviorally characterize all participants in comparable ways. Also, contrast participants were not selected and characterized comparably to ASD participants. Phenotyping has seldom been thorough, and when participants vary widely in age, types of assessments, and methods of ascertainment, the power to link iPSC model differences to the most important clinical reasons for study are nullified. Autism-critical social information is often shallow in iPSC studies to date. Thus, whether ASD iPSC differences relate to social differences or to other ASD characteristics has been largely unexamined.

Indirect evidence raises the hypothesis that the biological origin for social symptom severity in ASD is early embryogenesis. Greater social symptom severity in ASD toddlers is correlated with greater overactivity of three neurodevelopmental pathways (PI3K-AKT, RAS-ERK and WNT–β-catenin), and this overactivity (compared with neurotypical toddlers) occurs in 2D models of embryonic-stage ASD progenitors and neurons derived from ASD toddlers with enlarged brains [6]. Since these pathways are major regulators of cell cycle, proliferation, and maturation, their embryonic overactivity in ASD progenitors and neurons would be expected to increase proliferation and alter neuronal maturation, and in fact, that is what was found in the ASD toddler-derived cell models [9]. Missing is direct within-toddler evidence of correlations between embryonic ASD brain models and social symptom severity, and evidence of embryonic differences of ASD social subtypes.

In the present iPSC study, the 10 ASD participants were randomly drawn from our large cohort of ASD 1 to 4 year-olds toddlers who were extensively characterized using gold-standard ASD social symptom scores, social attention scores, social communication scores, language and cognitive IQ scores, and social and language brain measures (see Methods and Supplementary Materials). All ASD subjects were ascertained, recruited, phenotyped and tested using uniform procedures. This rigorous, robust and comprehensive method provided a large multimodality, multilevel normative ASD dataset for statistical comparisons.

Next, we selected brain cortical organoid size as our main measure of interest based on prior evidence and hypotheses. The a priori hypothesis was that variation in ASD cortical overgrowth starting in the first and second trimesters underlies social symptom severity in toddlers with ASD and is related to differences in  social brain, behavior, communication, and language ability in ASD at early postnatal ages. To test this, we generated and measured size growth of embryonic brain cortical organoids (BCOs) derived from iPSCs of the randomly selected but deeply socially characterized ASD toddlers and tested the correlation between BCO size and social symptom severity scores. To test the hypothesis that excess BCO growth in ASD is related to accelerated rates of proliferation and neuron differentiation, we measured this in BCOs with the most extreme enlargement.   To date, no ASD patient-derived iPSC study to date has examined whether variation in patient-derived embryonic BCO enlargement is correlated with variation in ASD social severity and ASD social subtypes at early ages. The question has high importance because if embryonic pathological cortical growth is correlated with later postnatal social severity and subtypes, then it means causal mechanisms driving to social severity and subtypes are already present before other speculated environmental and genetic factors associated with later phases of prenatal development. Put simply, it would indicate differential determinants of ASD social severity and subtypes are embryonic. This goes beyond the older question of whether or not ASD is prenatal, a question answered in the affirmative years ago based largely on computational and inferential evidence  [4–30]. This would change how ASD research designs must be formulated if progress is to be made on understanding how the central social symptoms of ASD come about.

Our choice of iPSC model and measurement was because early brain overgrowth, ascribed to excess excitatory neurons and first described two decades ago [11–14], is a characteristic found in many children with ASD. Further, it is among the most replicated findings in the idiopathic ASD neurobiology literature based on the largest meta-analysis and review to date by Persico and colleagues [31]. Their meta-analysis of 8310 ASD and control subjects from 44 MRI and 27 head circumference studies spanning toddlerhood to adulthood found highly significant brain and head circumference enlargement in ASD compared to controls. The most pronounced brain and head size increases occur at early ages in ASD [31], suggesting a prenatal origin. The first direct evidence of unusual prenatal brain development in ASD comes from ultrasound measures showing 2nd-trimester head enlargement in individuals later confirmed to have ASD [15]. However, no study has examined whether variation in patient-derived embryonic brain enlargement models are correlated with variation in an ASD child’s social severity.

Lastly, rather than a blind search for possible genes involved in ASD BCO growth, we examined a specific gene, *NDEL1*, because it is involved in embryonic neuronal proliferation and neurogenesis, neurite outgrowth [32], neuronal morphogenesis, migration and cell positioning. *NDEL1* shows patterned expression along anterior–posterior and dorsal–ventral axes in midgestational prenatal periods and contributes to cortical regionalization [33]. This cortical patterning effect is reduced in ASD postmortem cortex [34] and *NDEL1* is one among a set of implicated genes. Cerebral cortical size is affected by *NDEL1* regulation of mitotic spindle function [35]. Given these prominent justifications, we examined *NDEL1* expression in BCOs.

## Methods

The study was reviewed and approved by the UC San Diego Institutional Review Board, and all parents gave informed consent.

### Recruitment, diagnosis, and psychometric testing

In the present study, toddlers with ASD were not pre-selected based on brain size, clinical phenotype, or ASD risk gene status; instead, parents self-volunteered their child. From among > 400 ASD toddlers recruited at our Center using the *Get SET Early* method [36–38] and fully phenotyped between 2008 and 2011 as described previously [36], the parents of 23 toddlers agreed to participate in California Institute for Regenerative Medicine ASD program (https://www.cirm.ca.gov/). This program successfully reprogramed peripheral blood mononuclear cells to fully characterized iPSCs in 11 (9 M; 2F which is a 4.5:1 boy to girl ratio well-known in ASD) of the 23 toddlers by 2019 (Supplemental Table S3). The BCOs of 1 toddler did not grow, leaving a final set of 10 ASD toddlers from whom BCOs were successfully generated (Table 1). Licensed clinical psychologists blind to study hypotheses conducted evaluations of all toddlers using the Autism Diagnosis Observation Schedule (ADOS) [39], the Mullen Scales of Early Learning [40], and the Vineland Adaptive Behaviour Scales [41]. Diagnoses were based on DSM-V criteria [42], ADOS results and clinical judgement. Scores from the ADOS, Mullen and Vineland were used as measures of symptom severity, social, language and overall cognitive ability (Table 1). Clinical data from study ASD toddlers were compared with data from n = 1427 ASD and n = 475 TD toddlers tested at UCSD in the identical way.

**Table 1:** Summary of sex, age (range), and diagnostic and psychometric (means) characteristics of ASD toddlers

ASD subtype	Sex (M/F)	Age at 1st visit	ADOS total	IQ	Receptive language IQ	Expressive language IQ
Clinically severe	2/1	26 months (24–28)	22.0	54.6	45.3	43.6
Clinically mild	6/1	20 months (12–41)	13.7	97.0	92.0	92.2

The 10 ASD BCO toddlers were 1–3-years in age at intake (mean 1.8 years of age). This enabled within-child linkage of early-age ASD clinical and brain presentation with embryonic-stage BCO size. This is the first such ASD BCO study to examine such relationships within-child utilizing normative early age diagnostic scores, social and language abilities, social eye tracking, and regional neuroanatomic characteristics. Additionally, toddlers were longitudinally followed to confirm an ASD diagnosis and to obtain best estimate longitudinal clinical and social behavioral characteristics (Supplementary Material). Six control subjects, including 2 fully phenotyped typical toddlers, also participated.

### Eye tracking

Following standard procedures, a subset of ASD toddlers (N = 9) participated in eye tracking using a well-validated test designed to quantify levels of interest in social and non-social images [43–45]. These data were compared with data from N = 930 ASD and N = 357 TD toddlers tested in the identical way (Supplementary Materials).

### Brain imaging

Structural MRI images were collected on a subset of ASD toddlers (N = 9) using a 1.5 T GE scanner with traditional protocols [10, 46, 47]. Volumes for brain regions key for social and language development were calculated using FreeSurfer 5.3. These data were compared with data from N = 166 ASD and N = 100 TD toddlers MRI assayed in the identical way; analyses of these MRI datasets [46] show that by first factoring out overall brain size, differentially increased or decreased growth in different anatomic measures in ASD-relevant social, language, face processing and behavior regulation regions are isolated and highlighted in ASD toddlers (Supplementary Materials).

### Genetic analyses

All 10 ASD study toddlers were genetically assayed using targeted sequencing of a subset of 270 ASD risk genes by Eichler and colleagues [48, 49]. One ASD female among the 10 study ASD toddlers had a missense variant (p.Ser2390Phe, CADD score v1.0 equals 32) in *HECTD4*, which is a SFARI Level 1 ASD risk Gene [50]. This is a rare variant with the highest minor allele frequency < 0.01 observed in any population including 1000 Genomes Phase 3, ESP and gnomAD. The other 9 ASD toddlers were idiopathic having no ASD risk gene mutation.

### BCO generation in ASD and controls

**Fig. 1:**
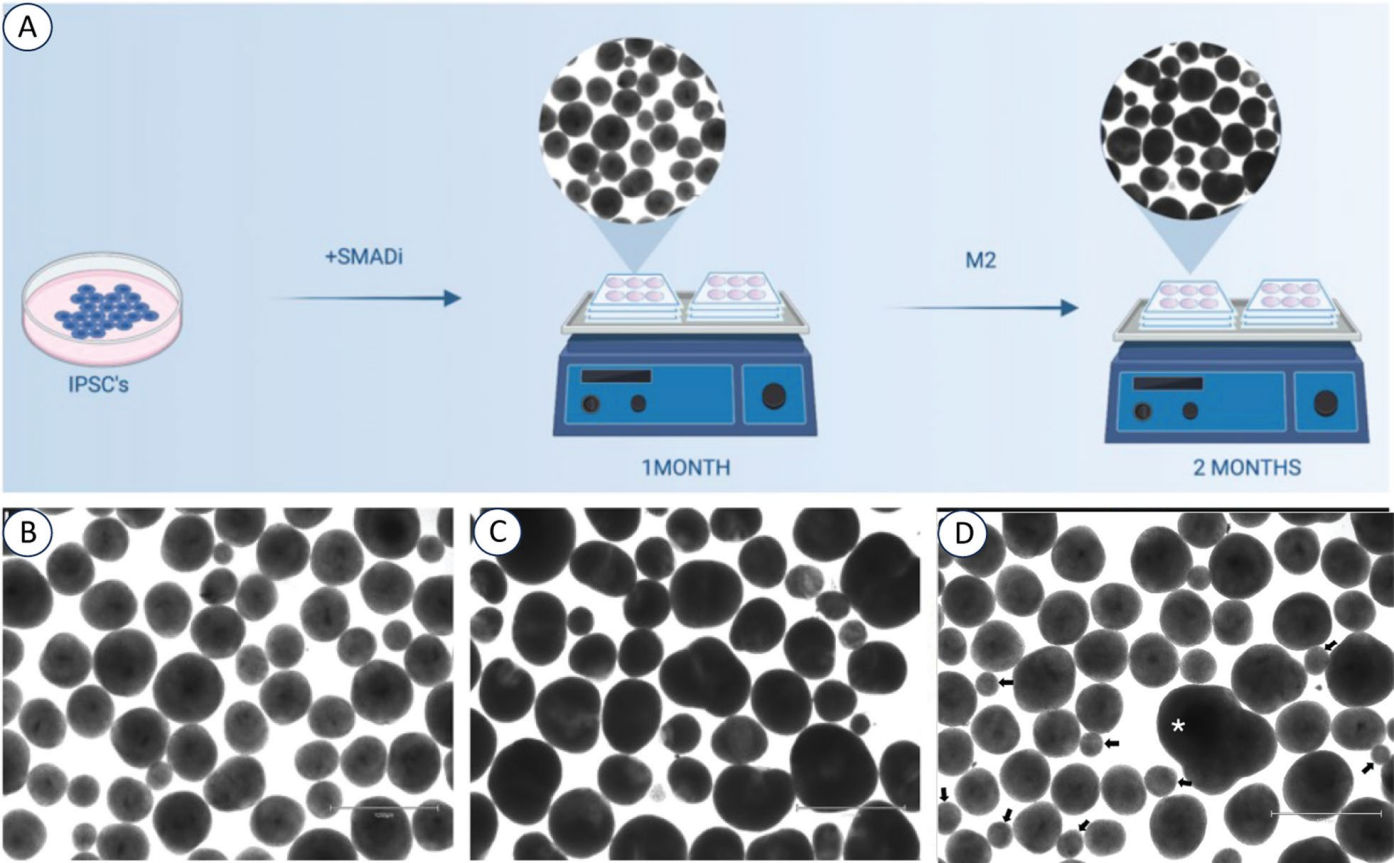
Culturing BCOs from human iPSCs. **A** Schematic representation of the experimental outline. IPSC-derived BCO were produced according to Trujillo et al. [1] in orbital shakers and measured at 1- and 2-months. Representative bright-field images of BCOs at 1-month (**B**) and 2-months (**C**), capturing both cell proliferation and differentiation phases during early embryogenesis. **D** Exclusion criteria were applied during the BCO measurement process; particular attention was paid to distinguishing genuine single organoids from fused organoids (asterisk) or spheres and non-organoid entities (small arrows). Small spherical structures (diameter < 200 μm; arrows) inconsistent with organoid morphology and potentially representing aggregates of necrotic cells, were excluded from the measurement. This criterion was applied uniformly across all images to maintain consistency in the data collected.

A robust and reproducible protocol developed in the Muotri lab [1] was used to generate BCOs from ASD and controls by iPSC technicians blind to clinical, behavioral and brain phenotypes and subtypes (Fig. 1; Supplementary Materials). They were generated in two independent batches in 2021 and 2022. BCOs were grown for 30 and 60 days, capturing neural progenitor cells and neurons during early embryogenesis (see Fig. 1). A full description of our organoid methods, including rigor and reproducibility, is now in *Nature Protocols* [51].

### BCO size replication in ASD and controls

To test overall replication of ASD vs control BCO size and growth effects (see **Results**), BCOs were generated in two independent batches, one in 2021 and the other in 2022. In the 2021 batch, BCOs from 10 ASD and 5 controls were generated, and, in 2022, BCOs from 6 of those 10 ASD toddlers and 3 of those 5 controls were generated again. A new typical toddler was also run in 2022, giving this second batch a total of 6 ASD and 4 controls. In both batches, BCOs were measured at 30 and 60 days.

### BCO measurement of ASD and controls

The diameters of organoids were measured from static images (Fig. 1). The process was designed to ensure accuracy and consistency of measurements across subjects (see Supplementary Materials). For the sampling and Imaging Protocol, we used three to six images obtained from each well at 1-month and 2-month time points post-organoid formation for each subject using Evos microscope M5000, objective 2X (ThermoFisher Scientific). These time points were chosen for their relevance to the developmental timeline of BCOs (proliferation and differentiation), for facilitating a comprehensive analysis of size variation over time, and for their approximate temporal overlap with our previous studies of ASD toddler-derived progenitors and neurons that differed from typicals on gene expression and cellular measures of cell cycle, proliferation and differentiation [6, 9]. The images were captured using a standardized imaging setup to maintain image quality and organoid visualization consistency. The diameter of all BCOs in every image were measured for a total of N = 2858 and N = 2052 individual BCOs in the 2021 and 2022 batches, respectively, which gave an overall total of 4910 individual BCOs from 15 subjects in 2021 and 10 subjects in 2022 giving an average of N = 98 BCOs measured per age point per subject.

The size measurement technique used the diameter of each organoid, which was measured utilizing the ImageJ software (version 2.14.0/1.54f), a widely recognized tool for image analysis in biological research. Measurements were conducted by drawing a line across the largest dimension of each organoid, ensuring that the measurement reflected the maximum diameter. This method was uniformly applied to all visible organoids within the image frame.

Exclusion criteria were applied during the measurement process; particular attention was paid to distinguishing genuine single organoids from fused organoids or spheres and non-organoid entities (eg., asterisk in Fig. 1D). Small spherical structures (diameter < 200 um) (eg, arrows Fig. 1D), inconsistent with organoid morphology and potentially representing aggregates of necrotic cells, were excluded from the measurement. These criteria were applied uniformly across all images to maintain consistency in the data collected.

### Comparative databases

Unique and key to the study design is that all social symptom, social and language clinical, cognitive, social attention, and social and language imaging data from ASD BCO toddlers were compared with comparably longitudinally phenotyped large samples of ASD and typical toddler norms ranging from N = 266 to N = 1902 toddlers who had been ascertained, recruited and phenotyped identically to the study toddlers. This enabled statistical analyses and interpretation of all ASD BCO data within the context of our large UCSD normative and ASD clinical, behavioral and imaging databases.

### NDEL1 activity and expression measurements

Using the 2022 batch of 6 ASD and 4 control BCOs, we measured NudE Neurodevelopment Protein 1 Like 1 (*NDEL1*) oligopeptidase activity in ASD and control BCOs because this enzyme activity plays roles in proliferation, cell division, neurogenesis, neurite outgrowth, and cell positioning and migration during embryogenesis and has been found to play roles in many other neuropsychiatric disorders [52]. In this 2022 batch, 2-month BCOs from the 6 ASD and 4 controls were collected and homogenized with buffer (50 mM Tris–HCl + 500 mM NaCl, pH 7.9) and centrifuged at 20,000 ×*g* for 10 min at 4 °C, and the supernatant was recovered and used for the Ndel1 enzyme activity measurements. Enzymatic assays were performed in a Tecan m1000 infinite microplate reader, using a 96-well flat black plate and at Ex = 320 nm and Em = 420 nm wavelengths. Ndel1 enzyme activity was measured by monitoring the hydrolysis of FRET substrate (Abz-GFSPFRQ-EDDnp) at 37 °C. For each reaction, BCO homogenate and buffer (NaCl 100 mM, 50 mM Tris–HCl, pH 7.4) containing 10 μM of FRET substrate were added, either in the absence or presence of 80 μM of chloranil (Ndel1 inhibitor [53]). Ndel1 specific activity was defined as the rate of hydrolysis in the absence of the inhibitor minus the rate measured in this inhibitor presence. Arbitrary Units of Fluorescence (AUF) values were obtained by the angular coefficient of each reaction, where 1 μM of substrate corresponds to 6047 AUF/s, using the m1000 infinite fluorimeter. Ndel1 activity was calculated using the formula: μM/min/mg = AUF/s × 60/mg of total protein concentration added to the reaction. The protein concentration of these samples was determined at 562 nm using the BCA method on a Nanodrop One.

We also performed real-time quantitative PCR to assess *NDEL1* expression. Total RNA was extracted from three distinct ASD BCO lines with larger sizes and three control BCOs using the RNeasy Mini Plus kit (Qiagen). Subsequently, 800 ng of total RNA were reverse transcribed into cDNA employing the Quantitect Reverse Transcription kit (Qiagen). For the assessment of *NDEL1* expression in BCOs, real-time quantitative PCR (RT-qPCR) was conducted using the RT^2^ SYBR Green Fluor qPCR mastermix (Qiagen) on a CFX Connect Real-Time PCR detection system with Maestro software (Bio-Rad; version 1.1). The PCR cycling parameters consisted of an initial denaturation at 95 °C for 10 min, followed by 40 cycles of 95 °C for 15 s and 60 °C for 1 min. Amplification and denaturation curves for all primers were meticulously examined to confirm the amplification of a single amplicon. Each RT-qPCR analysis was performed in triplicate. Normalization was achieved utilizing the endogenous control gene GAPDH, and the relative expression levels were calculated using the 2^−ΔΔCt^ method. Primers were obtained from IDT (Integrated DNA Technologies, Inc., Coralville, IA, USA), as PrimeTime qPCR primer assays, and employed the sequences previously validated in Li et al. [54]: GAPDH-F (5′-CCACGGCAAGTTCAACGGCACAG-3′), GAPDH-R (5′-GACGCCAGTAGACTCCACGACAT-3′), Ndel1-F (5′-AGCACCCGTTCATCACATCT-3′), and Ndel1-R (5′-GATGCTTGGCAGGAGCTTAGA-3′).

### Immunofluorescence assay

From the 2022 batch, N = 20 BCOs from the 2-month timepoint from 2 of the 6 ASD toddlers with the largest BCOs and N = 10 BCOs from one typical toddler were immunofluorescent assayed (Fig. 1f). These N = 30 BCOs were fixed in 4% paraformaldehyde (PFA) for 4 h at 4 °C, washed in PBS, dehydrated in 30% sucrose until they sank, and then embedded in O.C.T. compound (Sakura, Tokyo, Japan). The samples were sectioned at 20 µm using a cryostat. Sections were rehydrated, washed in PBS, and permeabilized with 0.1% Triton X-100 in PBS for 15 min. Blocking was performed with a solution of 0.1% Triton X-100 and 3% BSA in PBS for 2 h at room temperature. Sections were then incubated overnight at 4 °C with a mouse anti-NeuN primary antibody (ab1543P, 1:300) in a blocking solution. Following three washes in PBS, sections were incubated with Alexa Fluor 647-conjugated secondary antibody (Life Technologies, 1:1000) in a blocking solution. Nuclei were stained with DAPI for 5 min (1:10,000 in PBS). The slides were mounted with ProLong Gold antifade mountant (Life Technologies) and imaged using a Zeiss Z1 Axio Observer Apotome fluorescence microscope (Oberkochen, Germany). NeuN + and DAPI + cells were quantified across the entire section of each of 10 to 20 organoids per condition using a standardized ImageJ protocol. Although these experiments were not blinded, the quantification process was automated to minimize bias, applying identical settings and thresholds across all samples. Normalization was relative to DAPI-stained nuclei [55]. NeuN + /DAPI was calculated for each BCO. All measurements taken are provided in Supplemental Table S4.

### Statistical analyses

Mixed effect modelling using *lme4* and *lmerTest* packages in R and partial eta squared (${\eta }_{p}^{2}$) effect size [56, 57] was used to test a priori hypothesized mean BCO size differences between ASD and control groups and false discovery rate (FDR) was used to correct the *p*-values for multiple pairwise comparisons; a Pearson correlation to examine the relationship of BCO size by ASD symptom score; and Mann–Whitney U to test ASD clinical subtype differences in symptom severity. IQ, social behavior, and cortical region size of ASD toddlers were compared with typical and ASD normative data. Additionally, analyses of replicability of BCO growth and measurement were performed within-subject using the 2052 BCOs measured in the 2022 batch.

## Results

### ASD BCO overgrowth: greater BCO size in ASD than controls in both 2021 and 2022 batches

**Fig. 2:**
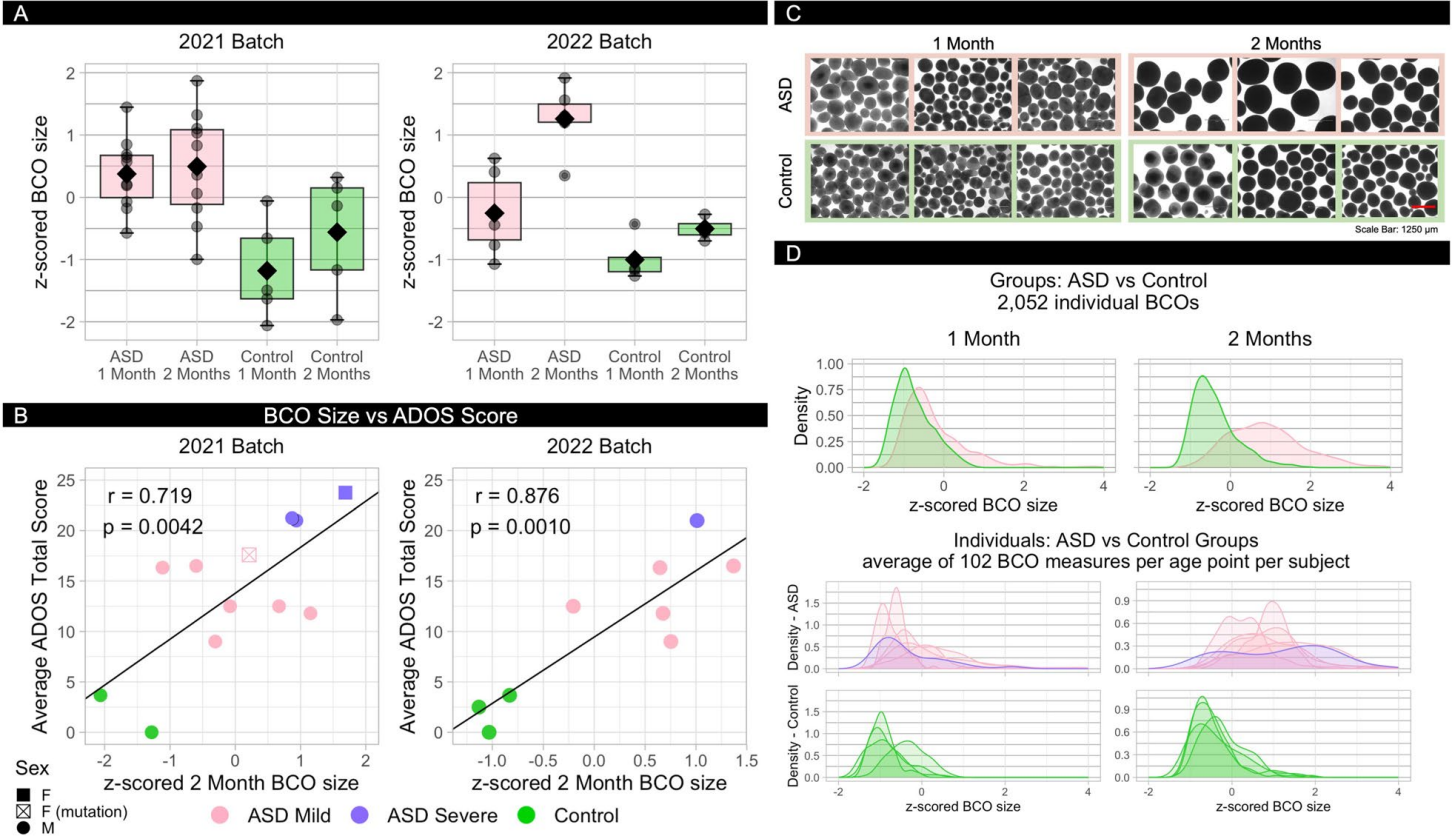
Comparing ASD and Control BCO size distributions and BCO size correlation with ADOS social symptom severity. **A** Average normalized BCO sizes for ASD and Control subjects at 1 and 2 months for each batch. **B** Correlation of normalized 2 month BCO size with ADOS Total score (SA + RRB), averaged across intake and longitudinal clinical assessment, for each batch (r = Pearson’s correlation). **C** Representative bright-field images of 3 ASD and 3 Control toddlers at 1 month and 2 months (scale bar: 1250 microns). **D** Density distributions of individual BCO sizes for all ASD vs all Control BCOs (top) and within subject (bottom), with an average of 102 BCOs per subject per age point

A linear mixed-effect model with random intercepts of subjects was fit to the data to examine the relationship between BCO size, group, and BCO timepoint effects. Controlling for BCO time point, analyses of the 2021 batch showed the ASD group mean was 39% larger than the control group mean and had a very large effect size (ASD > control size; $\beta$ = − 229.86, *t*(15) =  − 3.74, *p* = 0.002, ${\eta }_{p}^{2}$ = 0.48). Controlling for group, the average of 2-month BCOs was higher than the average of 1-month BCOs (2-month BCOs > 1-month; $\beta$ = 19.04, *t*(2846) = 2.81, *p* = 0.005, ${\eta }_{p}^{2}$ = 0.003). Figure 2A shows the 2021 batch ASD and control group mean BCO sizes based on the average BCO of each subject as well as BCO size for each subject at both BCO time points.

In the 2022 batch, ASD BCOs were 41% larger than control BCOs at 1- and 2-months (Fig. 2A). In fact, at 2-months, every ASD BCO was larger than every control (ASD: 1053, 1227, 1233, 1249, 1301, 1375 microns; and control: 837, 864, 884, 925 microns), and, while BCO sizes in controls increased by 14% between 1- and 2-months, ASD BCO sizes increased by 35% (t(8) = 1.98, *p* = 0.043). Figure 2C shows representative images of BCOs from 3 ASD and 3 controls at 1-month and 2-months timepoints. Figure 2D shows density plots of the 2052 individual measures at 1-month and 2-months from the 6 ASD and 4 controls at the group level and individual subject level.

A linear mixed-effect model with random intercepts of subjects was fit to the data to examine the relationship between BCO size and group and time point effects. Results in Table 2 show a main effect of group and time point as well as the interaction effect of group by time point. Out of six possible pairwise comparisons resulting from the interaction effect, we kept four comparisons based on our planned contrasts and we corrected the *p*-values by FDR (Table 2). ASD group BCOs were significantly larger than control BCOs at 1-month (i.e., ASD, 1 m—Control, 1 m) and 2-months (i.e., ASD, 2 m—Control, 2 m) with large effect sizes of 0.38 and 0.79, respectively. In addition, both ASD and control groups’ BCOs significantly increased in size from first month to the second month with large effect size (0.26) for ASD and small effect size (0.03) for controls (Table 2).

**Table 2:** Group interaction pairwise comparisons

Contrast	Estimate	SE	df	t.ratio	Adj. *p* value	η_p_^2^
ASD, 1 m—Control, 1 m	148.63	51.40	13.71	2.89	0.012	0.38
ASD, 1 m—ASD, 2 m	-314.31	11.83	2044.14	-26.57	0.000	0.26
Control, 1 m—Control, 2 m	-100.13	13.80	2044.12	-7.25	0.000	0.03
ASD, 2 m—Control, 2 m	362.82	51.14	13.37	7.09	0.000	0.79

2022 Batch

m = month, *η*_*p*_^*2*^ = Partial Eta Squared

## The larger the ASD BCO, the more severe the ASD social symptoms

Larger ASD BCO sizes were positively correlated with greater ASD social symptom severity in both the 2021 and 2022 batches (Fig. 2B). This correlation between BCO size × ADOS social symptom score was significant at 1-month (Pearson r(10) = 0.602, *p* = 0.019; 95% CI [0.147, 1]) and at 2-months in the 2021 batch (Pearson r(10) = 0.719, *p* = 0.0042; 95% CI [0.247, 0.915]; see Fig. 2B). Those ASD toddlers with larger BCO sizes had significantly more severe symptom scores (22.0 ± 1.5 vs. 13.7 ± 3.1; z = − 2.286, *p* = 0.0222; standardized effect size, Z/√(n + n) = 0.72).

In the 2022 replication batch, BCO size was again significantly correlated with ADOS social severity score (at 2-months: Pearson r(7) = 0.876, *p* = 0.0010; 95% CI [0.5854, 1]) (Fig. 2B).

## The 2021 batch: Toddlers with enlarged BCOs had the most severe social symptoms, lowest IQ, least social eye tracking attention, and most extreme cortical volumes in social, language, face, and sensory regions

All 10 ASD toddlers were in the 2021 batch, enabling relationships within-toddler to be made between BCO size in severe vs mild ASD toddlers and their clinical, behavior, and brain measures.

### BCOs in severe ASD were larger than BCOs in mild ASD

Linear mixed-effect model with random intercepts of subjects was fit to the data at the ASD subtype level (*i.e.,* clinically severe ASD, clinically mild ASD, and control) to further examine the main group effect stated above, and results showed a main effect of ASD subtype and BCO time point, as well as the interaction between ASD subtype and BCO time point (Table 3). Out of 15 possible pairwise comparisons resulting from the interaction effect, we kept nine comparisons based on our planned contrasts and we corrected the *p*-values by false discovery rate (FDR). All the subtype BCOs were significantly different from each other at 1-month (*e.g.,* Mild ASD, 1 m—Severe ASD, 1 m) and 2-months (*e.g.,* Mild ASD, 2 m—Control, 2 m) with large effect sizes (Table 3). At both BCO time points, the severe ASD subtype had the largest BCOs, followed by the mild ASD subtype; controls had the smallest BCOs. Moreover, across time points, the severe ASD subtype BCOs (*i.e.,* profound ASD, 1 m—profound ASD, 2 m) increased, but the mild ASD subtype BCOs did not change significantly from the first to the second month (Table 3).

**Table 3:** Subtype interaction pairwise comparisons

Contrast	Estimate	SE	df	t.ratio	Adj. *p* value	η_p_^2^
Mild ASD, 1 m—Severe ASD, 1 m	-151.22	69.95	19.18	-2.16	0.049	0.20
Mild ASD, 1 m—Control, 1 m	194.06	59.66	19.44	3.25	0.009	0.35
Severe ASD, 1 m—Control, 1 m	345.28	74.43	19.50	4.64	0.001	0.52
Mild ASD, 1 m—Mild ASD, 2 m	0.27	8.30	2846.56	0.03	0.974	-
Severe ASD, 1 m—Severe ASD, 2 m	-62.94	15.53	2846.08	-4.05	0.000	0.01
Control, 1 m—Control, 2 m	-49.37	17.79	2862.60	-2.78	0.010	0.00
Mild ASD, 2 m—Severe ASD, 2 m	-214.43	70.15	19.45	-3.06	0.010	0.32
Mild ASD, 2 m—Control, 2 m	144.42	60.69	21.18	2.38	0.034	0.21
Severe ASD, 2 m—Control, 2 m	358.84	75.34	20.74	4.76	0.000	0.52

2021 Batch

m = month, *η*_*p*_^*2*^ = Partial Eta Squared

### The severe ASD subtype with enlarged BCOs had severe social symptoms and very low social attention, low global IQ, and low receptive and expressive language

**Fig. 3:**
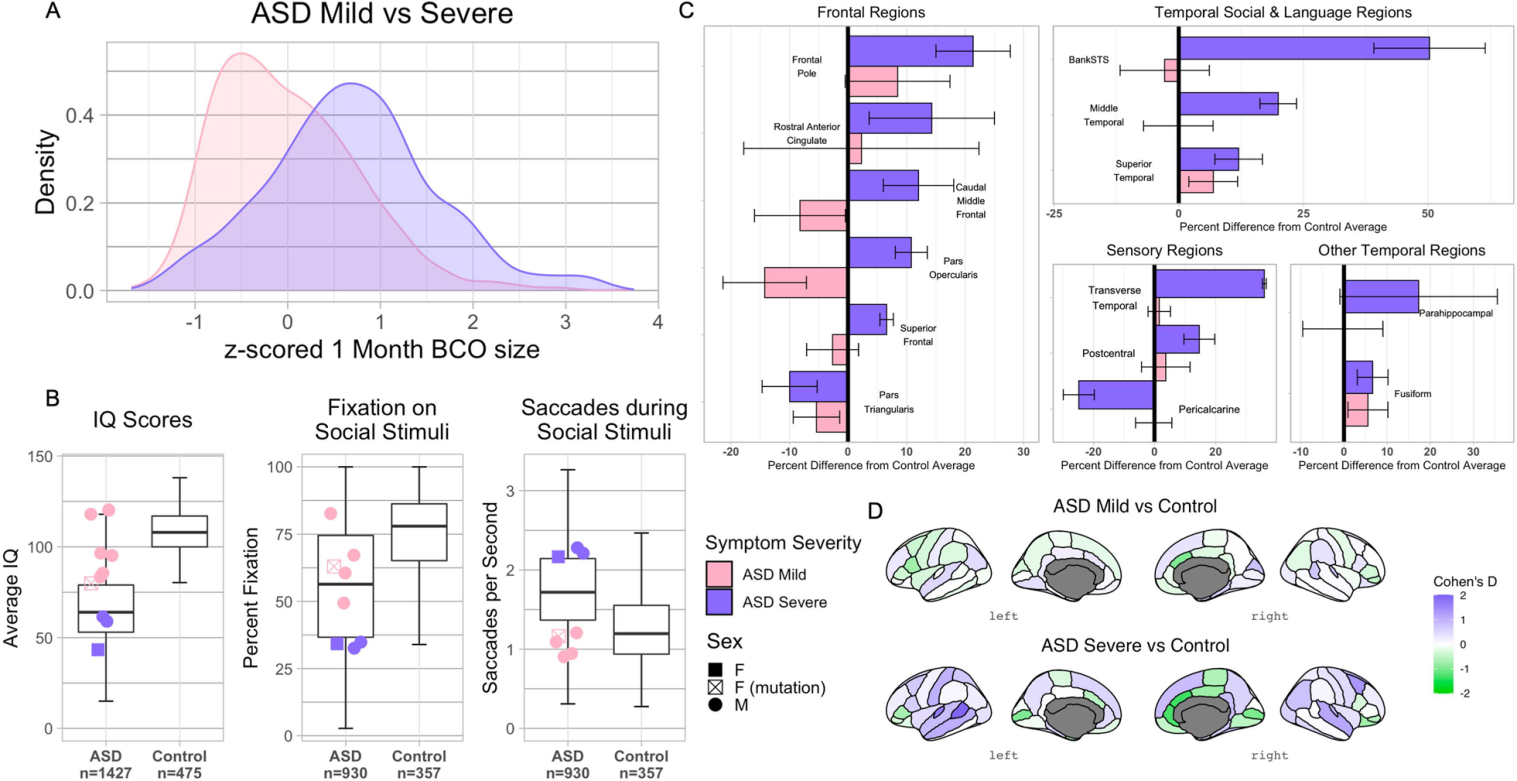
Subtype analysis of ASD toddlers in the 2021 Batch. **A** Density distribution of 953 individual BCO measurements for Clinically Mild and Clinically Severe ASD subtypes. **B** Shows performance of Mild and Severe ASD toddlers on IQ and social eye tracking attention measures compared to the UCSD ASD and Control toddler sample norms. The IQ norms are from N = 1427 ASD and N = 475 TD individuals; the norms for eye tracking Fixation on Social Stimuli and Saccades during Social Stimuli are from N = 930 ASD and N = 357 TD individuals. The average IQ scores of Mild and Severe ASD toddlers were calculated using Mullen, WISC, and WPPSI scores across multiple longitudinal visits. The IQ scores of the Severe ASD toddlers were considerably below neurotypical and even below the mean of our sample of 1427 ASD toddlers, while mild ASD toddlers had IQ scores in the neurotypical range. Social interest and attention were indexed by percent fixation on social stimuli, and saccades per second quantified the subject was  watching social stimuli during the eye tracking test. The Clinically Mild subtype showed eye tracking scores in the near typical range, while the Clinically Severe subtype fell in the lowest-performing quartile of ASD toddlers for both fixation and saccade measures [45]. **C** Regional MRI-based brain volume differences between subtypes and norms based on N = 100 typically developing toddlers [46]. Percent difference of Mild and Severe ASD subtype from the Control average volume for cortical areas involved in social and language processing and for auditory, visual, and somatosensory cortices in the left hemisphere. **D** Effect sizes of brain volume differences between ASD subtypes and Control norms, mapped to the corresponding cortical regions (Supplementary Materials)

Table 1 shows IQ scores for the 10 ASD toddlers *vs* norms from our large UCSD cohort of TD and ASD toddlers whose ages are comparable to the ASD toddlers in this BCO study. Figure 3 also shows measures of social interest and attention as indexed by the GeoPref social eye-tracking test in our 10 ASD toddlers vs norms from our large UCSD sample of TD and ASD toddlers. Figure 3A and Table 1 show that Clinically Severe ASD toddlers have (i) extremely large BCO sizes (ii) severe social symptoms, (iii) markedly low IQ and language, and (iv) low social eye-tracking interest and attention. Specifically, Table 1 and Fig. 3B show their social symptoms averaged 22 on the ADOS; global IQ scores were between − 2 standard deviations (SDs) and − 4 SDs (average IQ 54); receptive and expressive language scores were more than − 3.66 SD below average; and social visual attention eye-tracking scores were − 2.5 SD or lower. Permutation analyses of this Severe autism subtype *vs* UCSD TD norms were significantly different for IQ scores (*p* <  < 0.0001) and eye tracking scores (%fixation, *p* = 0.002; saccades/sec, *p* = 0.008). This Severe subtype had extremely large BCO sizes that were 102% greater than controls at 1-month and 69.8% at 2-months (permutation *p* = 0.008; *z* = 945, *p* = 0.025). The clinical profile of these Severe ASD toddlers is consistent with profound autism [3].

Figure 3B and Table 1 also show the other 7 ASD BCO toddlers had mild ASD clinical characteristics: Compared to our TD norms, they had only mildly lower IQ (96.6) and social eye-tracking attention. They also had mild-to-moderate ADOS scores averaging 13.7. This Clinically Milder subtype had BCO sizes that were 61.8% greater than controls at 1-month 1 and 21.9% at 2-months (permutation *p* = 0.006; *z* = 1480, *p* = 0.014).

### The severe ASD subtype with enlarged BCOs also had the greatest volume differences in social, language, face, and sensory cortices

The Severe/profound autism BCO subtype had cortical growth extremes in primary sensory cortices (Fig. 3C), receptive and expressive language cortices (Fig. 3C), and several social cortices (Fig. 3C) as compared with typical control average. Fig. 3D shows effect sizes of brain volume differences between ASD severe and mild subtypes and typical control norms, mapped to the corresponding regions. In some regions, overgrowth was 20–50% above neurotypicals (bank of the STS; middle temporal cortex; primary auditory cortex), while in visual cortex, growth was reduced by > 25% (Fig. 3C). Interestingly, pars triangularis, which is normatively involved in semantic and gestural processing, was also smaller than typical (Fig. 3C).

Importantly, as shown in Fig. 3C and D, these differences from the typical average generally exceeded those of the Clinically Milder BCO toddlers, who also showed an array of lesser differences from the typical average. For example, the Clinically Milder ASD toddlers did not differ from neurotypical in sensory and receptive language regions, but in left expressive language cortex they had growth differences opposite to Clinically Severe BCO toddlers, namely reduced volume of pars opercularis and caudal middle frontal gyrus, possibly suggesting a different functional impact on expressive language development in these two BCO subtypes. Both BCO subtypes had reduced left cortex size in semantic and gestural processing regions.

## Ndel1 oligopeptidase activity and expression correlation with BCO size in the 2022 batch

**Fig. 4:**
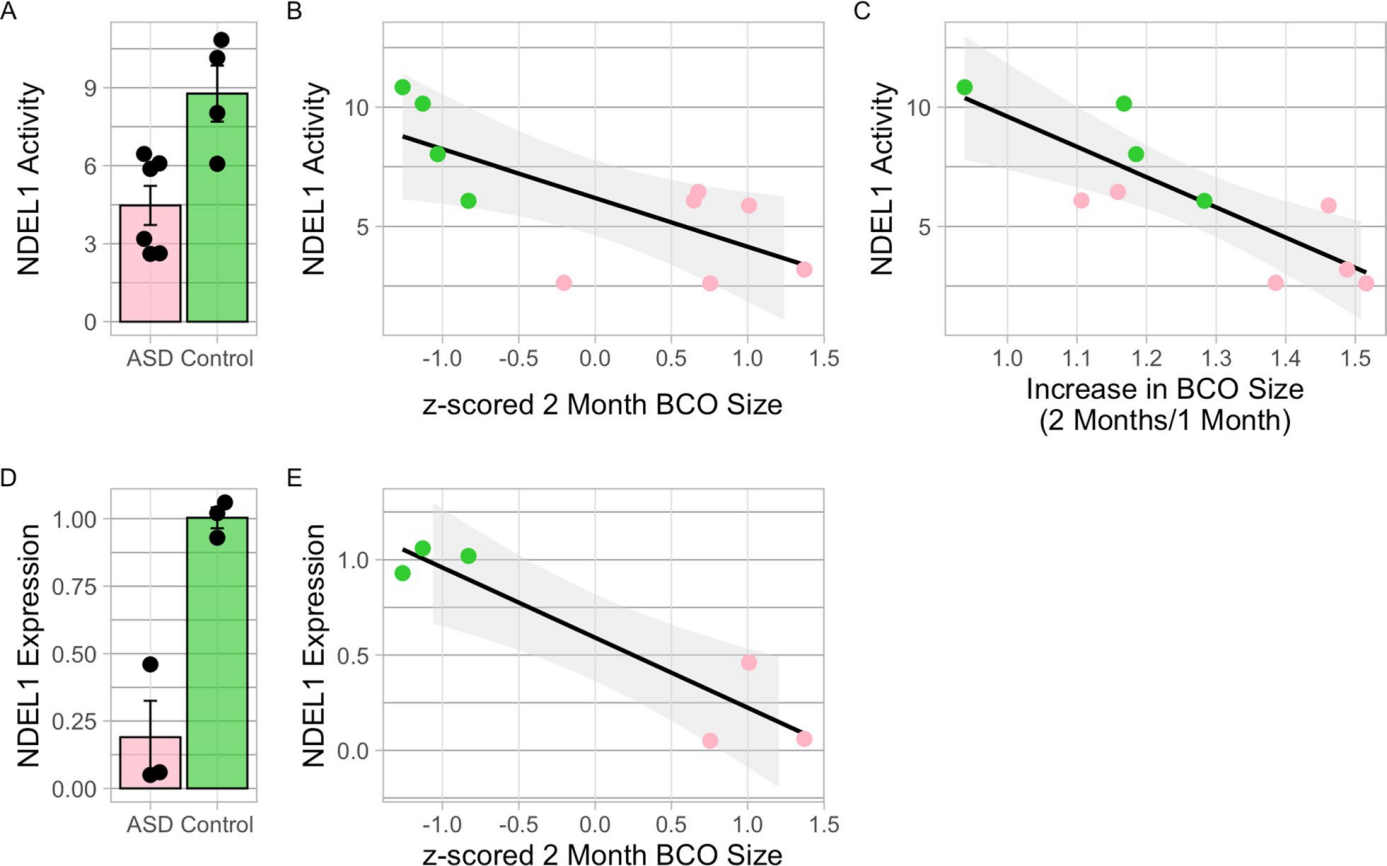
NDEL1 Oligopeptidase Activity and Expression in 2-Month-Old BCOs from Control and ASD BCOs. **A** NDEL1 activity exhibited a significant reduction in ASD compared to control BCOs. NDEL1 activity was assessed in freshly prepared sample homogenates and is presented as uM/min/mg of total protein. **B** BCO sizes at 2 months showed a significant negative correlation with NDEL1 activity, demonstrating an inverse relationship between this enzyme activity and BCO size (Pearson r(10) =  − 0.697; *p* = 0.025). **C** Growth rate of organoids also exhibited a significant negative correlation with NDEL1 activity (Spearman ρ(10) =  − 0.757; *p* = 0.014). **D** *NDEL1* expression was significantly downregulated in the 2-month-old ASD BCOs compared to controls. Expression levels were determined using the 2^−ΔΔCT^ method based on PCR data. **E** BCO sizes at 2 months also exhibited a significant negative correlation with *NDEL1* expression (Pearson r(6) =  − 0.932; *p* = 0.006)

The mean values for Ndel1 enzyme activity in the control and ASD BCOs were determined as 8.77 ± 2.16 and 4.48 ± 1.84 μM/min/mg, respectively, indicating a significant reduction in Ndel1 activity within BCOs derived from ASD cells (Student’s t-test (t = − 3.38, df = 8, *p* = 0.009, d = − 2.14)) (Fig. 4A). Furthermore, a notable inverse correlation emerged between Ndel1 activity and BCO size (Pearson r(10) =  − 0.697; *p* = 0.025; 95% CI [− 0.1201, − 0.9220]) (Fig. 4B), but Ndel1 was not significantly correlated with symptom severity. Interestingly, a negative correlation was also observed between Ndel1 activity and the growth rate of organoids between the first and second month (Spearman ρ(10) =  − 0.757; *p* = 0.014; 95% CI [− 0.1210, − 0.9131]) (Fig. 4C), indicating that Ndel1 lower activity corresponds to higher growth rate.

To explore potential alterations in *NDEL1* expression levels, we also conducted real-time PCR analysis. Our findings confirmed a significant downregulation of *NDEL1* expression in the 2-month-old BCOs compared to controls (Student’s t-test (t = − 5.79, df = 4, *p* = 0.004)) (Fig. 4D). Additionally, a significant negative correlation was observed between *NDEL1* expression and BCO size (Pearson r(6) = − 0.932; *p* = 0.006; 95% CI [− 0.4992, − 0.9928]) (Fig. 4E).

## Immunofluorescence assay: NeuN + and DAPI + Cell quantification in largest ASD BCOs versus control BCOs

**Fig. 5:**
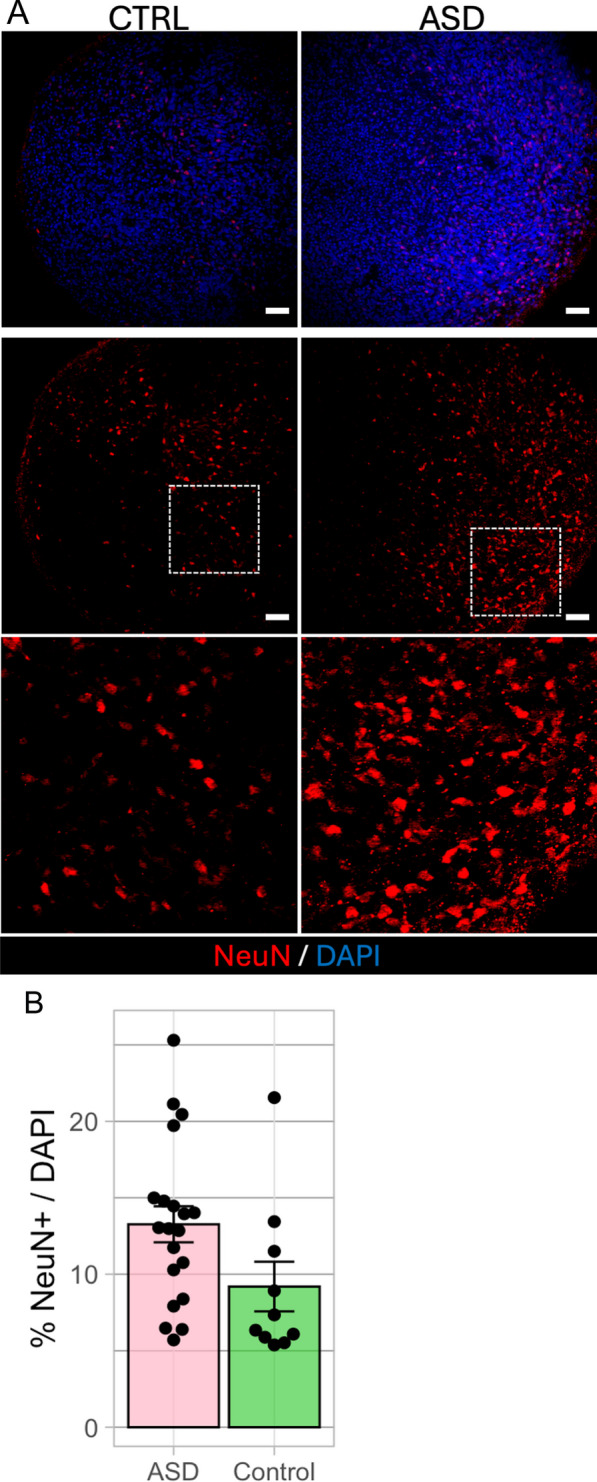
Immunofluorescence Assay: NeuN + and DAPI + Cell Quantification in Largest ASD BCOs vs Control BCOs. **A** Shows images of organoid sections from a control (left panels) and an ASD toddler with one of the largest BCO sizes (right panels) at 2-months stained for DAPI (upper panels) and NeuN^+^ mature neurons (lower panels.). White boxes are locations of magnified images for later quantification of percentage NeuN^+^/DAPI. **B** Box plot shows significantly greater percentage of NeuN + cells/DAPI in N = 20 individual BCOs from ASD toddlers vs N = 10 individual BCOs from a control. The complete set of NeuN + values is in Supplemental Table S4 and demonstrates consistent differences between ASD and control organoids across multiple independent experiments. The significant increase in NeuN + cells in ASD at 2-months during maturation phase compared to control, indicates ASD organoid enlargement may be due to accelerated differentiation or greater survival of neurons generated from neural progenitor cells. Data are %NeuN + /DAPI mean ± SEM. Mann–Whitney test, *p* = 0.0255). Scale bar 50 um

Figure 5A shows immunofluorescence images of organoid sections at 2-months stained for NeuN^+^ mature neurons in a control toddler and an ASD toddler with one of the largest BCO sizes. The box plot in Fig. 5B shows significantly greater %NeuN + cells/DAPI in N = 20 individual BCOs from two ASD toddlers versus N = 10 individual BCOs from a control toddler (Mann–Whitney test, *p* = 0.0255). This significant increase in ASD NeuN + cells at 2-months indicates ASD organoid enlargement may be due to accelerated differentiation or greater survival of neurons generated from neural progenitor cells.

## Discussion

By embryogenesis, the biological bases of two contrasting subtypes of ASD social and brain development—profound/severe autism and mild autism—are already distinguishable  and measurable and involve dysregulated proliferation and accelerated neurogenesis and growth. The larger the embryonic BCO size, the more severe the ASD toddler’s social symptoms; the more reduced the IQ, social attention, and language ability; and the more atypical the social, language, and sensory brain growth. Between embryonic ages 1- and 2-months, BCO growth was accelerated in ASD, being almost three times the control group, and those with the most embryonic-age overgrowth had accelerated neurogenesis. BCO growth rate and outcome size at 2-months were both highly correlated with reduced activity and expression of Ndel1, a gene known to govern embryonic proliferation, neurogenesis, and growth. In sum, accelerated proliferation, neurogenesis, and overgrowth of BCOs were extreme in profound ASD predicting severe social and brain outcomes, whereas milder BCO enlargement occurs in milder ASD. Thus, this work shows that the biological determinants of who will have mild autism and who will have profound autism, involve dysregulation of embryonic mechanisms that govern the rate and degree of cell proliferation, neurogenesis, and growth. These embryonic differences highlight the need to separate these contrasting ASD subtypes in iPSC experiments and develop subtype-specific treatments for idiopathic ASD toddlers.

Our findings reveal a significant inverse correlation between the growth rate of ASD organoids and Ndel1 activity. Ndel1, predominantly located in the centrosome, serves as a prime target for cell cycle-activated kinases. Phosphorylation of Ndel1 by the DYRK2-GSK3β complex is crucial for neuronal morphogenesis [58], while Aurora-A-mediated Ndel1 phosphorylation is essential for centrosomal separation, maturation, and mitotic entry [59]. Our observation of ASD toddler-derived organoids at 2-months in age displaying a higher count of mature neurons, suggests that accelerated growth/early maturation might stem from alterations in the dynamic of the cell cycle machinery, consequently affecting post-translational modifications of Ndel1. These modifications are highly likely to regulate its affinity with several binding partners [60], thereby also impacting Ndel1 activity levels.

Consistent with a long history of clinical, eye tracking and neuroimaging evidence of a minimum of two distinct ASD subgroups [8, 44, 45, 47, 61–67], the present BCO data also identified two ASD embryonic subtypes with distinctly different BCO growth, molecular, social, cognitive and social and language cortex growth phenotypes, a first in the field. These distinct embryonic precursors of the ASD mild and profound social subtypes are already present by embryogenesis. ASD toddlers with very enlarged BCOs had the most severe social ASD symptoms, reduced social attention indexed by eye tracking, reduced IQ and language, and atypical social, language and sensory cortical growth, while those with mild ASD symptoms and typical-level language and IQ measures had moderately enlarged BCOs. The constellation of neural, behavior, and clinical characteristics in those with extremely large BCOs were distinctive and significantly different from our large samples of typically toddlers who were ascertained, tested, and analyzed at our Center in the exactly the same way. Thus, determinants of a persistently severe profound subtype of ASD occur in embryogenesis and include accelerated neurogenesis. Notably, these toddlers with profound autism and enlarged BCOs had substantially enlarged primary auditory and somatosensory cortices, alongside reducedvisual cortices, a finding that highlights and helps explain sensory and social attention issues in ASD [43, 44, 67, 69–72]. Future larger sample BCO studies will undoubtedly reveal still other subtypes, given new gene expression and fMRI evidence [66].

To our knowledge, the present design is unique among ASD iPSC studies (Supplemental Tables S1 and S2) because it incorporated methods to statistically link iPSC-based measures of pathobiology to differences in ASD social communication, social attention and interest, and social and language ability and brain development. Remarkably, ASD-patient derived iPSC studies to date commonly ignore whether model system findings are correlated with the actual social developmental characteristics of the living ASD child whose iPSCs are being quantified. Yet, the point of looking at mechanisms *is* to explain what leads to the development of core ASD symptoms and challenges. To get to explanations, insight and clinical impact about the disorder, future iPSC designs must incorporate the same degree of rich information about the living ASD child within the context of the iPSC model. Our study is one example of how this can be done.

Thus, future ASD-derived iPSC designs should be within-subject which enables statistical linking of ASD organoid molecular and cellular biology to ASD social phenotypes. Designs should have uniform ASD and neurotypical subject ages, ascertainment, recruitment, diagnostic assessment, and psychometric assessment at early ages. To discover the embryonic mechanisms driving early-age social challenges in ASD, deeply phenotyped ASD infants and toddlers should be the subjects of iPSC studies. In addition to using the widely validated ADOS to index variation in severity of social symptoms at these early ages, designs should more deeply assess social variation using validated measures of social attention; social and language brain activity; social, language and sensory cortex growth; and in vivo gene expression including gene networks known to be correlated with social measures [6].

We showed the reproducibility of these BCO size and growth effects and used thousands of technical replicates in parallel to enhance rigor and reproducibility. Interestingly, whereas the narrow density plots of BCO size in controls suggest typical embryonic development is under tightly regulated genetic programs (Fig. 2D), those of ASD subjects had broader and more varied distributions of BCO sizes (Fig. 2D) consistent with a loss of such regulation and compatible with the common finding of ASD risk genes being regulatory and involved in early prenatal processes [5, 25].

Our past imaging-genomics work on ASD toddlers indicates that prenatally-relevant functional genomic mechanisms may be key to differentiating ASD severity subtypes [47, 73]. That work identified *NDEL1* as one of the pivotal hub genes within a gene co-expression module that is strongly associated with more severe clinical and imaging phenotypes, including surface area, cortical thickness, and functional neural responses to social speech [47, 73]. In the present study, lower *NDEL1* expression and activity was related to accelerated rate of BCO growth, overall BCO size, and accelerated neurogenesis. Other studies report Ndel1 is involved in multiple embryonic processes and in several neuropsychiatric disorders, in which lower activity was associated with the pathological condition [74, 75]. This encompasses roles in embryonic neuronal proliferation, neurogenesis, neurite outgrowth, neuronal morphogenesis, migration, and cell positioning. Dysregulation of these processes due to atypical *NDEL1* expression may result in altered cortical patterning and regionalization, as observed in animal models [76] and brain organoids [77].

A long-standing theory of ASD is that prenatal cell cycle pathobiology causes ASD early brain overgrowth, neural functional differences and ultimately leads to ASD social symptoms [4, 5, 7, 8, 12, 14]. Reviews of ASD postmortem, iPSC neural progenitor, neuron and organoid models, animal model and in vivo gene expression studies support this theory [4, 5] Other work  also supports the view that this pathobiology is related to ASD early brain overgrowth [8–10, 12, 47], which is among the most replicated findings in the idiopathic ASD neurobiology literature based on meta-analysis and review of 44 MRI and 27 head circumference studies to date [31]. However, missing from ASD-derived iPSC studies to date including the most recent ASD organoid study of molecular dysregulation [68], is statistical demonstration of the relevance of prenatal findings to ASD social communication differences. The present within-ASD subject design is the first to directly bridge the gap between an embryonic ASD pathobiological attribute and profound and mild ASD social symptoms at early ages.

## Limitations

With larger ASD social and BCO sample sizes, additional ASD BCO subtypes may be detected, as recent fMRI and MRI studies would predict. Also needed are comprehensive molecular and cellular BCO experiments to further delineate mechanisms causing variation in BCO enlargement in ASD as well as what causes the much wider within-child variation in BCO size as compared with the neurotypical BCOs. The genetic causes and cellular consequences of decreased Ndel1 activity and expression correlated with ASD BCOs enlargement remain to be specified. A limitation of most previous ASD patient-derived iPSC-based models is lack of within-subject statistical linkage of ASD molecular and cellular findings with variation in ASD social phenotypes. Without this, future ASD iPSC reports will continue to have limited impact on our understanding of the genetic, molecular and cellular mechanisms that cause the development and variation in the central feature of ASD: social affect and communication.

## Conclusions

The BCO overgrowth in our ASD toddlers has clinically meaningful interpretations because of the context of our large normative control and ASD data all collected in a uniform way [36, 37, 44–47], something lacking in all prior work with ASD cellular models (Supplemental Tables S1 and S2). ASD toddlers who had substantial BCO enlargement, accelerated neurogenesis and dysregulated Ndel1, were clinically identified at between 12 and 24 months of age and received state-of-the-art early ASD treatment. Despite this, their clinical characteristics did not substantially improve across time, a reminder of the challenges in addressing the outcomes of dysregulated embryonic processes. Thus, there is a need to re-imagine goals for future prevention and treatment research, particularly for profound ASD subtypes.

The biological determinants of who will have mild autism and who will have profound autism, involve dysregulation of embryonic mechanisms that govern the rate and degree of cell proliferation, neurogenesis, and growth. Those with the most accelerated cortical organoid growth and size at that extremely early developmental age, have severe social symptom, language, cognitive, social attention, and social and language brain growth outcomes. This sets the developmental mark that we must target to prevent profound autism.

### Supplementary Information

Additional file 1. 

Additional file 2. 

Additional file 3. 

Additional file 4.

Additional file 5. 

## Data Availability

Brain cortical organoid size data files and de-identified clinical scores for subjects are available one year after manuscript publication by contacting the first author.

## References

[1] Trujillo CA (2019). Complex oscillatory waves emerging from cortical organoids model early human brain network development. Cell Stem Cell.

[2] Bal VH, Kim SH, Fok M, Lord C (2019). Autism spectrum disorder symptoms from ages 2 to 19 years: implications for diagnosing adolescents and young adults. Autism Res.

[3] Lord C (2022). The Lancet Commission on the future of care and clinical research in autism. Lancet.

[4] Courchesne E, Pramparo T, Gazestani VH, Lombardo MV, Pierce K, Lewis NE (2019). The ASD living biology: from cell proliferation to clinical phenotype. Mol Psychiatry.

[5] Courchesne E, Gazestani VH, Lewis NE (2020). Prenatal origins of ASD: the when, what, and how of ASD development. Trends Neurosci.

[6] Gazestani VH (2019). A perturbed gene network containing PI3K-AKT, RAS-ERK and WNT-beta-catenin pathways in leukocytes is linked to ASD genetics and symptom severity. Nat Neurosci.

[7] Courchesne E (2007). Mapping early brain development in autism. Neuron.

[8] Courchesne E (2011). Neuron number and size in prefrontal cortex of children with autism. JAMA.

[9] Marchetto MC (2017). Altered proliferation and networks in neural cells derived from idiopathic autistic individuals. Mol Psychiatry.

[10] Pramparo T (2015). Cell cycle networks link gene expression dysregulation, mutation, and brain maldevelopment in autistic toddlers. Mol Syst Biol.

[11] Courchesne E (2001). Unusual brain growth patterns in early life in patients with autistic disorder: an MRI study. Neurology.

[12] Courchesne E (2002). Abnormal early brain development in autism. Mol Psychiatry.

[13] Courchesne E, Carper R, Akshoomoff N (2003). Evidence of brain overgrowth in the first year of life in autism. J Am Med Assoc.

[14] Courchesne E, Pierce K (2005). Brain overgrowth in autism during a critical time in development: implications for frontal pyramidal neuron and interneuron development and connectivity. Int J Dev Neurosci.

[15] Bonnet-Brilhault F (2018). Autism is a prenatal disorder: evidence from late gestation brain overgrowth. Autism Res.

[16] Stoner R (2014). Patches of disorganization in the neocortex of children with autism. N Engl J Med.

[17] Willsey AJ (2013). Coexpression networks implicate human midfetal deep cortical projection neurons in the pathogenesis of autism. Cell.

[18] Parikshak NN (2013). Integrative functional genomic analyses implicate specific molecular pathways and circuits in autism. Cell.

[19] Packer A (2016). Neocortical neurogenesis and the etiology of autism spectrum disorder. Neurosci Biobehav Rev.

[20] Kaushik G, Zarbalis KS (2016). Prenatal neurogenesis in autism spectrum disorders. Front Chem.

[21] Krishnan A (2016). Genome-wide prediction and functional characterization of the genetic basis of autism spectrum disorder. Nat Neurosci.

[22] Donovan AP, Basson MA (2017). The neuroanatomy of autism—a developmental perspective. J Anat.

[23] Grove J (2019). Identification of common genetic risk variants for autism spectrum disorder. Nat Genet.

[24] Gazestani VH, Cheng AWT, Courchesne E, Lewis NE. Autism genetics perturb prenatal neurodevelopment through a hierarchy of broadly-expressed and brain-specific genes. bioRxiv. 2020.

[25] Satterstrom FK (2020). Large-scale exome sequencing study implicates both developmental and functional changes in the neurobiology of autism. Cell.

[26] Pinto D (2010). Functional impact of global rare copy number variation in autism spectrum disorders. Nature.

[27] De Rubeis S (2014). Synaptic, transcriptional and chromatin genes disrupted in autism. Nature.

[28] O'Roak BJ (2012). Sporadic autism exomes reveal a highly interconnected protein network of de novo mutations. Nature.

[29] Yuen RKC (2017). Whole genome sequencing resource identifies 18 new candidate genes for autism spectrum disorder. Nat Neurosci.

[30] Courchesne E, Pierce K (2005). Why the frontal cortex in autism might be talking only to itself: local over-connectivity but long-distance disconnection. Curr Opin Neurobiol.

[31] Sacco R, Gabriele S, Persico AM (2015). Head circumference and brain size in autism spectrum disorder: a systematic review and meta-analysis. Psychiatry Res.

[32] Youn YH, Pramparo T, Hirotsune S, Wynshaw-Boris A (2009). Distinct dose-dependent cortical neuronal migration and neurite extension defects in Lis1 and Ndel1 mutant mice. J Neurosci.

[33] Li M (2018). Integrative functional genomic analysis of human brain development and neuropsychiatric risks. Science.

[34] Gandal MJ (2022). Broad transcriptomic dysregulation occurs across the cerebral cortex in ASD. Nature.

[35] Feng Y, Walsh CA (2004). Mitotic spindle regulation by Nde1 controls cerebral cortical size. Neuron.

[36] Pierce K (2019). Evaluation of the diagnostic stability of the early autism spectrum disorder phenotype in the general population starting at 12 months. JAMA Pediatr.

[37] Pierce K (2021). Get SET early to identify and treatment refer autism spectrum disorder at 1 year and discover factors that influence early diagnosis. J Pediatr.

[38] Pierce K (2011). Detecting, studying, and treating autism early: the one-year well-baby check-up approach. J Pediatr.

[39] Lord C, Luyster RJ, Gotham K, Guthrie W (2012). Autism diagnostic observation schedule, second edition (ADOS-2) manual (part II): toddler module.

[40] Mullen EM (1995). Mullen scales of early learning.

[41] Sparrow S, Cicchetti D, Balla D (2005). Vineland-II scales of adaptive behavior: survey form manual.

[42] Association AP. Diagnostic and statistical manual of mental disorders. 5th edn. 2013. 10.1176/appi.books.9780890425596

[43] Pierce K, Conant D, Hazin R, Stoner R, Desmond J (2011). Preference for geometric patterns early in life as a risk factor for autism. Arch Gen Psychiatry.

[44] Pierce K, Marinero S, Hazin R, McKenna B, Barnes CC, Malige A (2016). Eye tracking reveals abnormal visual preference for geometric images as an early biomarker of an autism spectrum disorder subtype associated with increased symptom severity. Biol Psychiatry.

[45] Wen TH (2022). Large scale validation of an early-age eye-tracking biomarker of an autism spectrum disorder subtype. Sci Rep.

[46] Duan K, et al. Differences in regional brain structure in toddlers with autism are related to future language outcomes. Nat Commun. 2024. 10.1038/s41467-024-48952-4 PMC11176156 38871689

[47] Lombardo MV (2021). Atypical genomic cortical patterning in autism with poor early language outcome. Sci Adv.

[48] Stessman HA (2017). Targeted sequencing identifies 91 neurodevelopmental-disorder risk genes with autism and developmental-disability biases. Nat Genet.

[49] Wang T (2020). Large-scale targeted sequencing identifies risk genes for neurodevelopmental disorders. Nat Commun.

[50] SFARI-Gene-Scoring (2019) https://gene-archive.sfari.org/database/human-gene/

[51] Fitzgerald MQ (2024). Generation of “semi-guided” cortical organoids with complex neural oscillations. Nat Protoc.

[52] Rodriguez B, Nani JV, Almeida PGC, Brietzke E, Lee RS, Hayashi MAF (2020). Neuropeptides and oligopeptidases in schizophrenia. Neurosci Biobehav Rev.

[53] Nani JV (2023). Identification of an ex vivo inhibitor of the schizophrenia biomarker Ndel1 by high throughput screening. Biochem Pharmacol.

[54] Li W (2021). Expression and function of Ndel1 during the differentiation of neural stem cells induced by hippocampal exosomesticle. Stem Cell Res Ther.

[55] Adams JW (2023). Impact of alcohol exposure on neural development and network formation in human cortical organoids. Mol Psychiatry.

[56] Bates D, Mächler M, Bolker B, Walker S (2015). Fitting Linear mixed-effects models using lme4. J Stat Softw.

[57] Kuznetsova A, Brockhoff PB, Christensen RHB (2017). lmerTest package: tests in linear mixed effects models. J Stat Softw.

[58] Woo Y (2019). Sequential phosphorylation of NDEL1 by the DYRK2-GSK3beta complex is critical for neuronal morphogenesis. Elife.

[59] Mori D (2007). NDEL1 phosphorylation by Aurora-A kinase is essential for centrosomal maturation, separation, and TACC3 recruitment. Mol Cell Biol.

[60] Garrott SR, Gillies JP, Siva A, Little SR, El Jbeily R, DeSantis ME (2023). Ndel1 disfavors dynein-dynactin-adaptor complex formation in two distinct ways. J Biol Chem.

[61] Courchesne E (1994). Cerebellar hypoplasia and hyperplasia in infantile autism. Lancet.

[62] Nordahl CW (2011). Brain enlargement is associated with regression in preschool-age boys with autism spectrum disorders. Proc Natl Acad Sci USA.

[63] Lombardo MV (2015). Different functional neural substrates for good and poor language outcome in autism. Neuron.

[64] Lombardo MV (2019). Default mode-visual network hypoconnectivity in an autism subtype with pronounced social visual engagement difficulties. Elife.

[65] Agelink van Rentergem JA, Deserno MK, Geurts HM (2021). Validation strategies for subtypes in psychiatry: a systematic review of research on autism spectrum disorder. Clin Psychol Rev.

[66] Xiao Y (2022). Neural responses to affective speech, including motherese, map onto clinical and social eye tracking profiles in toddlers with ASD. Nat Hum Behav.

[67] Pierce K (2023). Level of attention to motherese speech as an early marker of autism spectrum disorder. JAMA Netw Open.

[68] Jourdon A (2023). Modeling idiopathic autism in forebrain organoids reveals an imbalance of excitatory cortical neuron subtypes during early neurogenesis. Nat Neurosci.

[69] Leekam SR, Nieto C, Libby SJ, Wing L, Gould J (2007). Describing the sensory abnormalities of children and adults with autism. J Autism Dev Disord.

[70] Gomot M, Blanc R, Clery H, Roux S, Barthelemy C, Bruneau N (2011). Candidate electrophysiological endophenotypes of hyper-reactivity to change in autism. J Autism Dev Disord.

[71] Gomot M, Wicker B (2012). A challenging, unpredictable world for people with autism spectrum disorder. Int J Psychophysiol.

[72] Clery H, Bonnet-Brilhault F, Lenoir P, Barthelemy C, Bruneau N, Gomot M (2013). Atypical visual change processing in children with autism: an electrophysiological study. Psychophysiology.

[73] Lombardo MV (2018). Large-scale associations between the leukocyte transcriptome and BOLD responses to speech differ in autism early language outcome subtypes. Nat Neurosci.

[74] Gadelha A (2013). Plasma Ndel1 enzyme activity is reduced in patients with schizophrenia—A potential biomarker?. J Psychiatr Res.

[75] Dal Mas C (2019). Oligopeptidases activity in bipolar disorder: Ndel1 and angiotensin I converting enzyme. J Affect Disord.

[76] Sasaki S (2005). Complete loss of Ndel1 results in neuronal migration defects and early embryonic lethality. Mol Cell Biol.

[77] Ye F (2017). DISC1 regulates neurogenesis via modulating kinetochore attachment of Ndel1/Nde1 during mitosis. Neuron.

