# Supplementary material for: Embryonic origin of two ASD subtypes of social symptom severity: the larger the brain cortical organoid size, the more severe the social symptoms
Source: Mol Autism. 2024 May 25;15:22. doi: 10.1186/s13229-024-00602-8 (PMC11127428; doi:10.1186/s13229-024-00602-8)
Supplement: Supplementary file 1 — Additional file 1. [file 13229_2024_602_MOESM1_ESM.pdf]

Table S1: Idiopathic ASD patient-derived iPSC studies

ASD patient	ASD Sample Size	iPSC->?	Correlation w/ Clinical Data	scRNAseq	Reference	PMID	Link
Idiopathic	›	Organoids	No	Yes	Jourdan et al Nature Neuroscience 2023		https://doi.org/10.1038/s41593-023-01399-0
Idiopathic	8	NPC, Neurons	No	No	Marchetto et al. Molecular Psychiatry 2017	27378147	https://doi.org/10.1038%2Fmp.2016.95
Idiopathic	8	Neurons	No	No	Linker et al. Molecular Autism 2020	32591005	https://pubmed.ncbi.nlm.nih.gov/32591005/
Idiopathic & Syndromic	5 & 2	Astrocytes	No	No	Mansur et al. Int J Mol Sci. 2021	34299197	https://pubmed.ncbi.nlm.nih.gov/34299197/
Idiopathic	8	NPCs, Neurons	No	No	Li et al. Genes 2021	34946850	https://pubmed.ncbi.nlm.nih.gov/34946850/
Idiopathic	8	NPCs, Neurons	No	No	Ding et al. Am J Transl Res. 2021	34540009	https://pubmed.ncbi.nlm.nih.gov/34540009/
Idiopathic & Syndromic	4 & 3	NPC	No	No	Gomes et al. Front Neurosci. 2022	35360153	https://pubmed.ncbi.nlm.nih.gov/35360153/
Idiopathic & Syndromic	3 & 2	NPC	No	No	Connacher et al. Stem Cell Reports. 2022	35623351	https://pubmed.ncbi.nlm.nih.gov/35623351/
Idiopathic	1	Neurons	No	No	Song et al. Mol Brain. 2019	31699123	https://pubmed.ncbi.nlm.nih.gov/31699123/
Idiopathic	2	iPSC	No	No	Guo et al. Stem Cell Res. 2021	34438162	https://pubmed.ncbi.nlm.nih.gov/34438162/
Idiopathic	3	Neurons, Astrocytes	No	No	Russo et al. Biol Psychiatry. 2018	29129319	https://pubmed.ncbi.nlm.nih.gov/29129319/
Idiopathic	3	Neurons	No	No	Grunwald et al. Transl Psychiatry. 2019	31358727	https://pubmed.ncbi.nlm.nih.gov/31358727/
Idiopathic	3	NPC	No	No	Moore et al. Stem Cells Int. 2019	31191687	https://pubmed.ncbi.nlm.nih.gov/31191687/
Idiopathic	3	Neurons	No	No	Liu et al. Mol Neurobiol. 2017	27356918	https://pubmed.ncbi.nlm.nih.gov/27356918/
Idiopathic	3	NPC	No	No	Wang et al. Cell Stem Cell. 2020	32004479	https://pubmed.ncbi.nlm.nih.gov/32004479/
Idiopathic	4	Organoids	No	No	Mariani et al. Cell. 2015	26186191	https://pubmed.ncbi.nlm.nih.gov/26186191/
Idiopathic	4	Organoids	No	No	Ilieva et al. Mol Psychiatry. 2022	35618886	https://pubmed.ncbi.nlm.nih.gov/35618886/
Idiopathic	4	Organoids	No	No	Ejlersen et al. J Neural Transm. 2022	35266053	https://pubmed.ncbi.nlm.nih.gov/35266053/
Idiopathic	5	iPSC	No	No	Suzuki et al. Molecular Psychiatry 2015	25582616	https://pubmed.ncbi.nlm.nih.gov/25582616/
Idiopathic	6	NPC	No	No	Sanchez-Sanchez et al. Hum Mutat. 2018	29969175	https://pubmed.ncbi.nlm.nih.gov/29969175/
Idiopathic	6	NPC, Neurons	No	No	DeRosa et al. Sci. Rep. 2018	29849033	https://pubmed.ncbi.nlm.nih.gov/29849033/
Idiopathic	6	NPC, Neurons	No	No	Griesi-Olivera et al. Mol Psychiatry. 2021	32060413	https://pubmed.ncbi.nlm.nih.gov/32060413/
Idiopathic	8	NPC, Neurons	No	No	Schafer et al. Nat. Neurosci. 2019	30617258	https://pubmed.ncbi.nlm.nih.gov/30617258/
Idiopathic	8	Neurons	No	No	Amatya et al. Stem Cell Reports. 2019	31474529	https://pubmed.ncbi.nlm.nih.gov/31474529/
Idiopathic	9	Neurons	No	No	Adhya et al. Biol Psychiatry. 2021	32826066	https://pubmed.ncbi.nlm.nih.gov/32826066/
Idiopathic & Syndromic	43 & 22	iPSCs	No	No	Brick et al. Stem Cells Transl Med. 2014	25273538	https://pubmed.ncbi.nlm.nih.gov/25273538/
Idiopathic & ASD traits	2 & 2	Neurons	No	No	Lewis et al. Mol Autism. 2019	31893020	https://pubmed.ncbi.nlm.nih.gov/31893020/
