# Supplementary material for: Embryonic origin of two ASD subtypes of social symptom severity: the larger the brain cortical organoid size, the more severe the social symptoms
Source: Mol Autism. 2024 May 25;15:22. doi: 10.1186/s13229-024-00602-8 (PMC11127428; doi:10.1186/s13229-024-00602-8)
Supplement: Supplementary file 2 — Additional file 2. [file 13229_2024_602_MOESM2_ESM.pdf]

Table S2. Syndromic patient-derived iPSC studies

ASD Sample Size	Source	Genes and CNVs of interest	iPSC > ?	Correlation w/ Clinical Data	scRNAseq	Reference	PMID
1 patient/gene	ASD patient-derived	13 genes, 1 CNV	Neurons	No	No	Deneault et al. Elife. 2019	30747104
3 & 2	1q21.1 del & dup patient-derived	1q21.1 del & dup	Neurons	No	No	Chapman et al. Mol Psychiatry. 2022	34112971
5	15q11-q13.1 dup	15q11-q13.1 dup	Neurons	No	No	Germain et al. Mol Autism. 2014	25694803
1	15q13.3 dup patient-derived	15q13.3 dup	NPCs, Neurons	No	No	Meganathan et al. BMC Biol. 2021	34320968
2 & 1	Angelman Syndrome and Prader-Willi Syndrom	15q11-q13 del (maternal or paternal)	NPCs, Neurons	No	No	Chamberlain et al. PNAS. 2010	20876107
1 & 1	16p11.2 del and dup patient-derived	16p11.2 del & dup	Organoids	No	No	Urresti et al. Mol Psychiatry. 2021	34548630
13	16p11.2 del patient-derived	16p11.2 del	NPCs	No	No	Roth et al. Elife. 2020	33169669
3 & 3	16p11.2 del and dup patient-derived	16p11.2 del & dup	Neurons	No	No	Deshpande et al. Cell Rep. 2017	29212016
15	22q11.2 del patient-derived	22q11.2 del	Organoids	No	No	Khan et al. Nat Med. 2020	32989314
2	Phelan-McDermid Syndrome	22q13.3 del	Neurons	No	No	Shcheglovitov et al. Nature. 2013	24132240
1	RELN & CACNA1H patient-derived	CACNA1H, RELN	NPCs	No	No	Teles E Silva et al. Transl Psychiatry. 2022	35668055
2	Timothy Syndrome patient-derived	Ca(v)1.2	Neurons	No	No	Paşca et al. Nat Med. 2011	22120178
2	Timothy Syndrome patient-derived	Ca(v)1.2	Cardiomyocytes	No	No	Yazawa et al. Nature. 2011	21307850
2	Timothy Syndrome patient-derived	Ca(v)1.2	Neurons	No	No	Krey et al. Nat Neurosci. 2013	23313911
1	Timothy Syndrome patient-derived	Ca(v)1.2	NPCs, Neurons	No	No	Tian et al. Genome Med. 2014	25360157
3	Timothy Syndrome patient-derived	Ca(v)1.2	Organoids	No	No	Birey et al. Nature. 2017	28445465
3	Timothy Syndrome patient-derived	Ca(v)1.2	Neurons	No	No	Panagiotakos et al. Elife. 2019	31868578
3	Timothy Syndrome patient-derived	Ca(v)1.3	Organoids	No	No	Birey et al. Cell Stem Cell. 2022	34990580
2	Rett Syndrome patient-derived	CDKL5	Neurons	No	No	Amenduni et al. Eur J Hum Genet. 2011	21750574
2	Rett Syndrome patient-derived	CDKL5	Neurons	No	No	Ricciardi et al. Nat Cell Biol. 2012	22922712
2	Rett Syndrome patient-derived	CDKL5	Neurons	No	No	Wu et al. Neurobiol Dis. 2022	36202289
3	CNTNAP2 patient-derived	CNTNAP2	Organoids	No	Yes	de Jong et al. Nat Commun. 2021	34471112
2	Rubenstein-Taybi patient-derived	CREBBP	Neurons	No	No	Alari et al. Int J Mol Sci. 2021	34071322
5	Rubenstein-Taybi patient-derived	CREBBP/EP300	Neurons	No	No	Calzari et al. Mol Neurobiol. 2020	32562237
1	Kleefstra patient-derived	EHMT1	Neurons	No	No	Nagy et al. Transl Psychiatry. 2017	28742076
3	Kleefstra patient-derived	EHMT1	Neurons	No	No	Frega et al. Nat Commun. 2019	31666522
8	Fragile X patient-derived	FMR1	NPCs, Neurons	No	No	Raj et al. Cell Rep. 2021	33852833
3	Fragile X patient-derived	FMR1	NPCs, Neurons	No	No	Sheridan et al. PLoS One. 2011	22022567
3	Fragile X patient-derived	FMR1	iPSCs	No	No	Urbach et al. Cell Stem Cell. 2010	20452313
3	Fragile X patient-derived	FMR1	Neurons	No	No	Doers et al. Stem Cells Dev. 2014	24654675
3	Fragile X patient-derived	FMR1	Neurons	No	No	Halevy et al. Stem Cell Reports. 2014	25483109
1	Female Fragile X premutation carrier	FMR1 premutation	Neurons	No	No	Liu et al. Hum Mol Genet. 2012	22641815
3	MBD5 patient-derived	MBD5	NPCs	No	No	Mullegama et al. Sci Rep. 2021	34050248
1	Rett Syndrome patient-derived	MECP2	Neurons	No	No	Hotta et al. Nat Methods. 2009	19404254
1	Rett Syndrome patient-derived	MECP2	NPCs and Neuron	No	No	Muotri et al. Nature. 2010	21085180
4	Rett Syndrome patient-derived	MECP2	Neurons	No	No	Marchetto et al. Cell 2010	21074045
5	Rett Syndrome patient-derived	MECP2	Neurons	No	No	Kim et al. Proc Natl Acad Sci U S A. 2011	21807996
3	Rett Syndrome patient-derived	MECP2	Neurons	No	No	Ananiev et al. PLoS One. 2011	21966470
4	Rett Syndrome patient-derived	MECP2	Neurons	No	No	Cheung et al. Hum Mol Genet. 2011	21372149
1	Rett Syndrome patient-derived	MECP2	Neurons	No	No	Nott et al. Nat Neurosci. 2016	27428650
3	Rett Syndrome patient-derived	MECP2	NPCs in 3D hydro	No	No	Zhang et al. Proc Natl Acad Sci U S A. 2016	26944080
1	Rett Syndrome patient-derived	MECP2	Neurons	No	No	Tang et al. Proc Natl Acad Sci U S A. 2016	26733678
1	Rett Syndrome patient-derived	MECP2	NPCs	No	No	Yoo et al. Biochem Biophys Res Commun.	29050935
2	Rett Syndrome patient-derived	MECP2	Neurons	No	No	Mellios et al. Mol Psychiatry. 2018	28439102
1	Rett Syndrome patient-derived	MECP2	NPCs and Neuron	No	No	Sena de Souza et al. Mol Neurobiol. 2019	31214863
2	Rett Syndrome patient-derived	MECP2	NPCs, Astrocytes,	No	No	Kim et al. PLoS One. 2019	30789962
2	Rett Syndrome patient-derived	MECP2	Organoids	No	No	Yildirim et al. Elife. 2022	35904330
3	Rett Syndrome patient-derived	MECP2	Neurons	No	No	Perego et al. Int J Mol Sci. 2022	36430969
3	Rett Syndrome patient-derived	MECP2	Neurons	No	No	Haase et al. Int J Mol Sci. 2022	36232428
3	MECP2 Duplication Syndrome patients	MECP2 dup	Neurons	No	No	Nageshappa et al. Mol Psychiatry. 2016	26347316
1 & 1	ASD patient-derived	MECP2, TRPC6	NPC, Neurons	No	No	Griesi-Olivera et al. Mol Psychiatry. 2015	25385366
1	Rett Syndrome patient-derived	MECP2E1	Neurons	No	No	Djuric et al. Neurobiol. Dis. 2015	25644311
3	ASD patient-derived	NRXN1A	Neurons	No	No	Avazzadeh et al. Mol Autism. 2019	31893021
3	ASD PTCHD1-AS patient-derived	PTCHD1-AS	Neurons	No	No	Ross et al. Biol Psychiatry. 2020	31540669
1	ASD RELN patient-derived and Single gene mutat	RELN	Neurons	No	No	Arioka et al. Transl Psychiatry. 2018	30022058
2	ASD SHANK2 patients	SHANK2	Neurons	No	No	Zaslavsky et al. Nat Neurosci. 2019	30911184
1	ASD SHANK2 patient	SHANK2	Neurons	No	No	Lutz et al. Front Mol Neurosci. 2021	34899182
3	ASD (SHANK3) patient-derived & Single gene mu	SHANK3	Organoids	No	Yes	Wang et al. Nat Commun. 2022	36202854
3	Noonan Syndrome patient-derived	SHP2	Organoids	No	Yes	Kim et al. Int J Mol Sci. 2022	36430334
3	Noonan Syndrome patient-derived	SHP2	Organoids, NPCs	No	No	Ju et al. Stem Cell Res Ther. 2020	32493428
5	Pitt-Hopkins patient-derived	TCF4	Organoids, NPCs,	No	Yes	Papes et al. Nat Commun. 2022	35501322
1	Tuberous Sclerosis patient-derived	TSC1	NPCs	No	No	Martin et al. Mol Autism. 2020	31921404
1	Tuberous Sclerosis patient-derived	TSC2	NPCs, Neurons, A	No	No	Li et al. Stem Cell Reports. 2017	28344003
