# Supplementary material for: Embryonic origin of two ASD subtypes of social symptom severity: the larger the brain cortical organoid size, the more severe the social symptoms
Source: Mol Autism. 2024 May 25;15:22. doi: 10.1186/s13229-024-00602-8 (PMC11127428; doi:10.1186/s13229-024-00602-8)
Supplement: Supplementary file 3 — Additional file 3. [file 13229_2024_602_MOESM3_ESM.pdf]

Supplement Table S4: Sex, ethnicity and race of the 11 ASD and 6 control study subjects.

Group	Sex M/F	Hispanic %	Race
ASD	9/2	32%	8 caucasian,<br>1 asian,<br>1 unknown
Control	6/0	0	6 caucasian
