# Supplementary material for: Embryonic origin of two ASD subtypes of social symptom severity: the larger the brain cortical organoid size, the more severe the social symptoms
Source: Mol Autism. 2024 May 25;15:22. doi: 10.1186/s13229-024-00602-8 (PMC11127428; doi:10.1186/s13229-024-00602-8)
Supplement: Supplementary file 5 — Additional file 5. [file 13229_2024_602_MOESM5_ESM.docx]

**Embryonic origin of two ASD subtypes of social symptom severity:**

**The larger the brain cortical organoid size, the more severe the social symptoms**

**Supplementary Material**

Eric Courchesne, Ph.D.^1**^, Vani Taluja. B.A. ^1^, Sanaz Nazari, Ph.D.^1*^, Ph.D., Caitlin M. Aamodt, Ph.D.^6^, Karen Pierce, Ph.D.^1^, Kuaikuai Duan, Ph.D. ^1^, Sunny Stophaeros, M.S. ^1^, Linda Lopez^1^, B.A., Cynthia Carter Barnes, Ph.D.^1^, Jaden Troxel, B.A. ^1^, Kathleen Campbell, M.D. ., M.H.Sc. ^1^, Tianyun Wang, Ph.D.^2,3^, Kendra Hoekzema^4^, Evan E. Eichler, Ph.D.^4,5^, Joao V. Nani, Ph.D.^6,7,^, Wirla Pontes^6^, Sandra Sanchez Sanchez^6^, Michael V. Lombardo, Ph.D. ^9^, Janaina S. de Souza, Ph.D.^6^, Mirian A. F. Hayashi, Ph.D.^7^, Alysson R. Muotri, Ph.D. ^6,8^ **

**Affiliations:**

^1^Autism Center of Excellence, Department of Neurosciences, University of California, San Diego, La Jolla, CA, USA

^2^ Department of Medical Genetics, Center for Medical Genetics, Peking University Health Science Center, Beijing 100191, China

^3^ Neuroscience Research Institute, Peking University; Key Laboratory for Neuroscience, Ministry of Education of China & National Health Commission of China, Beijing 100191, China

^4^ Department of Genome Sciences, University of Washington School of Medicine, Seattle, WA 98195, USA

^5^ Howard Hughes Medical Institute, University of Washington, Seattle, WA 98195, USA

^6^ Department of Pediatrics and Department of Molecular and Cellular Medicine, University of California, San Diego, La Jolla, CA, USA

^7^ Department of Pharmacology, Escola Paulista de Medicina (EPM), Universidade Federal de São Paulo (UNIFESP), SP, Brazil

^8^ Rady Children’s Hospital, Center for Academic Research and Training in Anthropogeny (CARTA), Kavli Institute for Brain and Mind, Archealization Center (ArchC), La Jolla, CA, USA

^9^ Laboratory for Autism and Neurodevelopmental Disorders, Center for Neuroscience and Cognitive Systems, Istituto Italiano di Tecnologia, Rovereto, Italy

^*^**Senior Biostatistician**

****Correspondence to:**

Eric Courchesne, Ph.D., [ecourchesne@health.ucsd.edu](mailto:ecourchesne@health.ucsd.edu), Department of Neurosciences, University of California, San Diego, 8110 La Jolla Shores Dr., La Jolla, CA 92037

Alysson Muotri, Ph.D., [muotri@health.ucsd.edu](mailto:muotri@health.ucsd.edu), Department of Pediatrics and Department of Molecular and Cellular Medicine, University of California, San Diego, Gilman Drive, La Jolla, CA 92093

**Clinical and Eye Tracking Data Analyses: Longitudinal analyses to get best estimate scores**

All study ASD toddlers were psychometrically and diagnostically tested twice and as many as on 5 separate clinical visits. For each toddler, we took the mean of Total (SA + RRB) scores collected from ADOS administration across visits to find an average longitudinal, best-estimate ADOS score for each subject. The average subject age during administration was 33.9 months (range=12.6 - 116.0, median= 30.7 months, sd = 1.3) and an average of 3.2 clinical visits were used across the 11 subjects. They were compared with 1,902 ASD and TD toddlers ascertained, recruited, and clinically diagnosed and psychometrically phenotyped in the same way in the same age range^1-3^.

Similarly, we averaged IQ and eye tracking data from 2 to up to 5 visits. The best-estimate IQ was calculated using the mean of all available Mullen ELC, WISC FSIQ, and WPPSI FSIQ scores across visits for each of the 1,902 subjects including the BCO subjects. For the eye tracking measures, percent fixation on social stimuli and saccades per second while attending to social stimuli were also calculated using the mean of available data across clinical visits, excluding any sessions with poor eye tracking quality due to lack of child cooperation or other technical issues. The average subject age during administration was 28.1 months (range=5.0 - 144.4 months, median=27.1 months, sd=1.0) and an average of 1.8 visits were used for each of the total 1,287 subjects who were tested in the social eye tracking attention paradigm, including the BCO subjects.

**Eye Tracking Methods: apparatus, stimuli, and procedures (see our published Methods ^3-5^)**

Eye-gaze data was collected from study ASD toddlers using the Tobii T120 (Tobii, Stockholm, Sweden; www. tobii. com; 60 Hz sampling rate; 1280 Å~ 1024) while toddlers watched ‘The Geo-Pref Test’ (62.22 s), which consisted of two rectangular areas of interest (AOIs, 525 Å~ 363 pixels) each containing dynamic geometric (DGI) or social images (DSI; social images used with permission from Gaiam Americas Inc., Copyright 2003, Gaiam Americas, Inc.), identical to stimuli used in our previous work^3-5^). To control for biases due to spatial location, side of stimulus presentation varied across subjects. To ensure that only the toddler’s gaze was tracked and free from parent influence, standardized instructions were read to parents. A five-point calibration was then performed using animated cartoon ducks with sounds, and data was only used if calibration results, determined via graphical output and verified via screenshots, fell within manufacturer-reported parameters (accuracy, 0.5 degrees).

Data was processed using Tobii Studio (Tobii Fixation Filter, velocity threshold: 35 ms/window). Total fixation

duration, fixation count within each AOI, and fixation duration within each AOI were exported and analyzed offline. Percent fixation duration/AOI was computed by dividing the total fixation duration within an AOI by the fixation duration across the entire video. N-1 total fixations/total fixation duration was used to calculate saccades/sec within each AOI. The ASD study toddlers were tested the same way as 1,287 ASD ASD and TD toddlers in the same age range^3^.

**MRI Methods: Acquisition, measurement, and normalization**

**MRI data acquisition and processing.** Imaging data were collected on a 1.5T General Electric MRI scanner during natural sleep at night; no sedation was used. Structural MRI (sMRI) data were collected with a T1-weighted IR-FSPGR (inversion recovery fast-spoiled prepared gradient recalled) sagittal protocol with TE (echo time) = 2.8 ms, TR (repetition time) = 6.5 ms, flip angle = 12°, bandwidth = 31.25 kHz, field of view = 24 cm, and slice thickness = 1.2 mm. All sMRI scans were parcellated using FreeSurfer 5.3 (<http://surfer.nmr.mgh.harvard.edu/>) ^6^ based on the Desikan-Killiany atlas ^7^ to provide global and regional brain morphometric measures, including total brain volume, total surface area (SA), mean cortical thickness, cortical sub-regional volume/SA/thickness, and subcortical volumes. FreeSurfer aligns each toddler’s brain to an average brain derived from cortical folding patterns through nonlinear surface-based registration ^8^. This tool has been validated for studies of children ^9^ and has shown great success in large pediatric studies ^10-12^. Quality evaluation was further performed on the raw and segmented sMRI scans by two independent raters (Kathleen Campbell and Michael Datko) with rating scale ranging from 0 to 3 (0=best, 1=great, 2=usable, 3=unusable). Out of 447 sMRI scans, 75 –including the MRI scan from one ASD BCO toddler– were rated as unusable and were excluded from the study, yielding 372 scans from 275 toddlers. Among 275 toddlers, 166 were ASD including 9 of the ASD BCO toddlers; 100 were typically developing (TD) toddlers. These 275 ASD and TD toddlers were used here.

**Developmental and gender effects adjustments and percent difference computation.** We estimated the developmental and sex effects on the brain using brain morphometric measures from 100 TD toddlers who passed quality control and showed no delay previously. We then regressed them out on all ASD (including the 9 ASD BCO toddlers) and TD subjects to adjust for age and sex effects ^13^. The resulting brain volume measures were then used to estimate regional mean volume of 100 TDs, and we then computed the percent difference of all 9 BCO ASD subjects from TD means for the brain regions of interest.

**Genetic Analyses: targeted gene sequencing using smMIPs**

ASD risk genes were targeted in N=864 UC San Diego ACE toddlers including the 10 ASD study toddlers using targeted DNA sequencing of the coding regions for sets of 270 and 125 ASD and neurodevelopmental disorder risk genes using single-molecule molecular inversion probes (smMIPs)^14,15^. These included >179 high confidence SFARI Level 1 and 2 genes. See full lists genes in Bao et al ^16^. One ASD female also in the present BCO study had a missense variant (p.Ser2390Phe, CADD score v1.0 equals 32) in HECTD4, which is a SFARI Level 1 ASD risk Gene^17^.

**Brain Cortical Organoids Methods**

A robust and reproducible protocol developed in the Muotri lab^18^ was used to generate the BCOs in orbital shakers from iPSCs of subjects by technicians blind to clinical, behavioral and brain phenotypes and subtypes. BCOs were generated in three independent batches and sampling was done at 1 month and 2 months. Despite executing these experiments in sequential months, the protocols employed for organoid generation were identical for each. It is acknowledged that inherent variabilities in brain organoid development can lead to main effects of different experimental batches. To mitigate these variabilities, control and ASD group organoids were concurrently cultivated and analyzed within each experiment. This approach ensures that any observed differences in organoid sizes reflect genuine biological differences between study groups rather than experimental variation. Notably, despite the main effect of Experiment, the comparative analysis consistently demonstrated that organoids from the ASD group exhibited larger sizes than those from the control group, reinforcing the reproducibility of our findings across different experimental sets. We initially decided to plot % differences, normalizing the size to control organoids, masking the batch differences. However, we realized that showing the actual data would be more transparent to the readers.

Feeder-free iPSCs were fed daily with mTeSR1 for 7 days. Colonies were dissociated using Accutase (Life Technologies) in PBS (1:1) for 10 minutes at 37 °C and centrifuged for 3 minutes at 150 x *g*. The cell pellet was resuspended in mTeSR1 supplemented with 10 μM SB431542 (SB; Stemgent, Cambridge, MA, USA) and 1 μM Dorsomorphin (Dorso; R&D Systems, Minneapolis, MN, USA). Approximately 4 × 10^6^ cells were transferred to one well of a 6-well plate and kept in suspension under rotation (95 rpm) in the presence of 5 μM ROCK inhibitor (Y-27632; Calbiochem, Sigma-Aldrich, St. Louis, MO, USA) for 24 hours to form free-floating spheres. After 3 days, mTeSR1 was substituted by Media1 [Neurobasal (Life Technologies) supplemented with GlutaMAX, 1% Gem21 NeuroPlex (Gemini Bio-Products), 1% N2 NeuroPlex (Gemini Bio-Products), 1% NEAA (Life Technologies), 1% PS (Life Technologies), 10 μM SB and 1 μM Dorso] for 7 days. Then, the cells were maintained in Media2 [Neurobasal with GlutaMAX, 1% Gem21 NeuroPlex, 1% NEAA and 1% PS] supplemented with 20 ng/mL FGF2 (Life Technologies) for 7 days, followed by 7 additional days in Media2 supplemented with 20 ng/mL of FGF2 and 20 ng/mL EGF (PeproTech, Rocky Hill, NJ, USA). Next, cells were transferred to Media3 [Media2 supplemented with 10 ng/mL of BDNF, 10 ng/mL of GDNF, 10 ng/mL of NT-3 (all from PeproTech), 200 μM L-ascorbic acid and 1 mM dibutyryl-cAMP (Sigma-Aldrich) to promote maturation, gliogenesis and activity]. After 7 days, cortical organoids were maintained in Media2 for as long as needed, with media changes every 3-4 days.

Representative bright-field images of BCOs at 1 and 2 month months captured both cell proliferation and differentiation phases during early embryogenesis, and organoid diameters were measured from >100 BCOs per age point per subject, as we did previously^18^. See Fig 1 in main text showing representative cross-section of a BCO showing the presence of neural progenitor cells (Sox2+) and neurons (Map2+). BCOs did not grow for 1 of the 11 ASD toddlers. BCOs were also generated from 5 control males.

**Results: Differences in BCO size ratios consistent across Experiments 1 and 2**

Variabilities in brain organoid development can lead to main effects of different experimental batches. In this study, absolute BCO sizes were different between Experiments 1 and 2 batches, but within Experiment ratios were quite similar and not significantly different: For example, for the 6 ASD and 3 control subjects who were in both experiments, the ratio of control BCO size vs ASD size was 0.73 (449 vs 614 microns) in Experiment 1 at one-month and 0.85 (786/929 microns) in Experiment 2 at one-month. For these 6 ASD patients who were in both Experiments, the BCO diameter ratio of Exp 1 vs Exp 2 was 0.66 (614 vs 929 microns), and for the 3 controls who were in both Experiments, the BCO diameter ratio of Exp 1 vs 2 was 0.57 (449 vs 786 microns). These ratio relationships were also found at two-months: for ASD in Exp 1 vs 2 the ratio is 0.49 and for control it is 0.47; and for control vs ASD in Exp 1 it is 0.68 and for control vs ASD in Exp 2 it is 0.71. Thus, the large increase in size in ASD BCO vs control was reproducible across experiments and months. Thus, despite the main effect of Experiment on absolute BCO size, the comparative analysis consistently demonstrated that organoids from the ASD group exhibited larger sizes than those from the control group, reinforcing the reproducibility of our findings across different experimental sets.

**REFERENCES**

1. Pierce K, Gazestani V, Bacon E, et al. Get SET Early to Identify and Treatment Refer Autism Spectrum Disorder at 1 Year and Discover Factors That Influence Early Diagnosis. *J Pediatr*. Apr 26 2021;doi:10.1016/j.jpeds.2021.04.041

2. Pierce K, Gazestani VH, Bacon E, et al. Evaluation of the Diagnostic Stability of the Early Autism Spectrum Disorder Phenotype in the General Population Starting at 12 Months. *JAMA Pediatr*. Jun 1 2019;173(6):578-587. doi:10.1001/jamapediatrics.2019.0624

3. Wen TH, Cheng A, Andreason C, et al. Large scale validation of an early-age eye-tracking biomarker of an autism spectrum disorder subtype. *Sci Rep*. Mar 11 2022;12(1):4253. doi:10.1038/s41598-022-08102-6

4. Pierce K, Conant D, Hazin R, Stoner R, Desmond J. Preference for geometric patterns early in life as a risk factor for autism. *Arch Gen Psychiatry*. Jan 2011;68(1):101-9. doi:10.1001/archgenpsychiatry.2010.113

5. Pierce K, Marinero S, Hazin R, McKenna B, Barnes CC, Malige A. Eye Tracking Reveals Abnormal Visual Preference for Geometric Images as an Early Biomarker of an Autism Spectrum Disorder Subtype Associated With Increased Symptom Severity. *Biol Psychiatry*. Apr 15 2016;79(8):657-66. doi:10.1016/j.biopsych.2015.03.032

6. Dale AM, Fischl B, Sereno MI. Cortical surface-based analysis. I. Segmentation and surface reconstruction. *Neuroimage*. Feb 1999;9(2):179-94. doi:10.1006/nimg.1998.0395

7. Desikan RS, Segonne F, Fischl B, et al. An automated labeling system for subdividing the human cerebral cortex on MRI scans into gyral based regions of interest. *Neuroimage*. Jul 1 2006;31(3):968-80. doi:10.1016/j.neuroimage.2006.01.021

8. Fischl B, Sereno MI, Dale AM. Cortical surface-based analysis. II: Inflation, flattening, and a surface-based coordinate system. *Neuroimage*. Feb 1999;9(2):195-207. doi:10.1006/nimg.1998.0396

9. Ghosh SS, Kakunoori S, Augustinack J, et al. Evaluating the validity of volume-based and surface-based brain image registration for developmental cognitive neuroscience studies in children 4 to 11 years of age. *Neuroimage*. Oct 15 2010;53(1):85-93. doi:10.1016/j.neuroimage.2010.05.075

10. Lombardo MV, Eyler L, Pramparo T, et al. Atypical genomic cortical patterning in autism with poor early language outcome *Science Advances*. 2021;

11. Jernigan TL, Brown TT, Hagler DJ, Jr., et al. The Pediatric Imaging, Neurocognition, and Genetics (PING) Data Repository. *Neuroimage*. Jan 1 2016;124(Pt B):1149-1154. doi:10.1016/j.neuroimage.2015.04.057

12. Levman J, MacDonald P, Lim AR, Forgeron C, Takahashi E. A pediatric structural MRI analysis of healthy brain development from newborns to young adults. *Hum Brain Mapp*. Dec 2017;38(12):5931-5942. doi:10.1002/hbm.23799

13. Duan K, Chen J, Calhoun VD, et al. Neural correlates of cognitive function and symptoms in attention-deficit/hyperactivity disorder in adults. *Neuroimage Clin*. 2018;19:374-383. doi:10.1016/j.nicl.2018.04.035

14. Stessman HA, Xiong B, Coe BP, et al. Targeted sequencing identifies 91 neurodevelopmental-disorder risk genes with autism and developmental-disability biases. *Nat Genet*. Apr 2017;49(4):515-526. doi:10.1038/ng.3792

15. Wang T, Hoekzema K, Vecchio D, et al. Large-scale targeted sequencing identifies risk genes for neurodevelopmental disorders. *Nat Commun*. Oct 1 2020;11(1):4932. doi:10.1038/s41467-020-18723-y

16. Bao B, Zahiri J, Gazestani VH, et al. A predictive ensemble classifier for molecular diagnosis of ASD at ages 1 to 4 years. *MedRxiv*. 2022;

17. SFARI-Gene-Scoring. <https://gene-archive.sfari.org/database/human-gene/>. 2019;

18. Trujillo CA, Gao R, Negraes PD, et al. Complex Oscillatory Waves Emerging from Cortical Organoids Model Early Human Brain Network Development. *Cell Stem Cell*. Oct 3 2019;25(4):558-569 e7. doi:10.1016/j.stem.2019.08.002
